# Super-resolution diffractive neural network for all-optical direction of arrival estimation beyond diffraction limits

**DOI:** 10.1038/s41377-024-01511-4

**Published:** 2024-07-10

**Authors:** Sheng Gao, Hang Chen, Yichen Wang, Zhengyang Duan, Haiou Zhang, Zhi Sun, Yuan Shen, Xing Lin

**Affiliations:** 1 Department of Electronic Engineering, Tsinghua University, Beijing, 100084 China; 2 Beijing National Research Center for Information Science and Technology, Tsinghua University, Beijing, 100084 China

**Keywords:** Sub-wavelength optics, Applied optics

## Abstract

Wireless sensing of the wave propagation direction from radio sources lays the foundation for communication, radar, navigation, etc. However, the existing signal processing paradigm for the direction of arrival estimation requires the radio frequency electronic circuit to demodulate and sample the multichannel baseband signals followed by a complicated computing process, which places the fundamental limit on its sensing speed and energy efficiency. Here, we propose the super-resolution diffractive neural networks (S-DNN) to process electromagnetic (EM) waves directly for the DOA estimation at the speed of light. The multilayer meta-structures of S-DNN generate super-oscillatory angular responses in local angular regions that can perform the all-optical DOA estimation with angular resolutions beyond the diffraction limit. The spatial-temporal multiplexing of passive and reconfigurable S-DNNs is utilized to achieve high-resolution DOA estimation over a wide field of view. The S-DNN is validated for the DOA estimation of multiple radio sources over 5 GHz frequency bandwidth with estimation latency over two to four orders of magnitude lower than the state-of-the-art commercial devices in principle. The results achieve the angular resolution over an order of magnitude, experimentally demonstrated with four times, higher than diffraction-limited resolution. We also apply S-DNN’s edge computing capability, assisted by reconfigurable intelligent surfaces, for extremely low-latency integrated sensing and communication with low power consumption. Our work is a significant step towards utilizing photonic computing processors to facilitate various wireless sensing and communication tasks with advantages in both computing paradigms and performance over electronic computing.

## Abstract (web summary)

A super-resolution diffractive neural network, featuring super-oscillatory angular responses, can perform all-optical DOA estimation with an angular resolution beyond the diffraction limit, which facilitates low-latency integrated sensing and communication.

## Graphical abstract

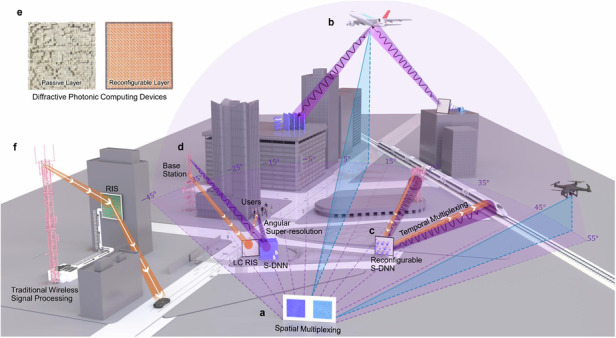

## Introduction

Wireless sensing and communication have become essential parts of modern life. The direction of arrival (DOA) estimation, i.e., the radio direction-finding, utilizing the array signal processing technique to retrieve the angular direction of electromagnetic (EM) field sources, is a critical technology and has facilitated broad applications in both civilian and military fields^1–3^. The conventional methods, e.g., the widely-used multiple signal classification (MUSIC) algorithms, require large numbers of radio frequency (RF) electronic circuits for acquiring multi-channel baseband signals before digital signal processing^1^. The high hardware and algorithm complexities and the massive data sampling hamper its performance in latency, power consumption, and cost. Therefore, it is imminent to develop new types of computing paradigms to process RF signals more effectively for DOA estimation beyond electronic processors^4,5^.

Recent research works on photonic processors have demonstrated their major advantages in computing speed, computing throughput, and energy efficiency^6–22^. By encoding RF signals in the optical domain and computing with photons, photonic processors can achieve functionalities of filtering^23^, temporal integration and differentiation^24^, and blind source separations with broader bandwidth^25,26^. To directly process the RF signals, diffractive neural networks^27–29^ and surface plasmonic neural networks^30^ were constructed, which modulate the EM waves and process its carried information for different tasks, e.g., object recognition and wireless codec, at the speed of light. Compared with surface plasmonic, the meta-structures in diffractive neural networks can modulate three-dimensional instead of two-dimensional EM waves, which enables the network to have higher scalability for large-scale spatial computing. Recently proposed meta-structures consist of an achromatic meta-lenses array, enabling intelligent depth measurement^31^. Nevertheless, the resolution of the existing system is still constrained by the diffraction limit, and its application for advanced wireless sensing tasks has not been explored. Besides, applying reconfigurable intelligent surfaces (RIS) to modulate the spatial EM waves and construct the next generation of communication systems^32–36^ lacks perception and computing capabilities. Thus, RIS necessitates communicating with the base station to receive the control signals and users’ angular directions^37,38^, which makes it challenging to provide low-latency communication services for high-speed rail and autopilot.

**Fig. 1:**
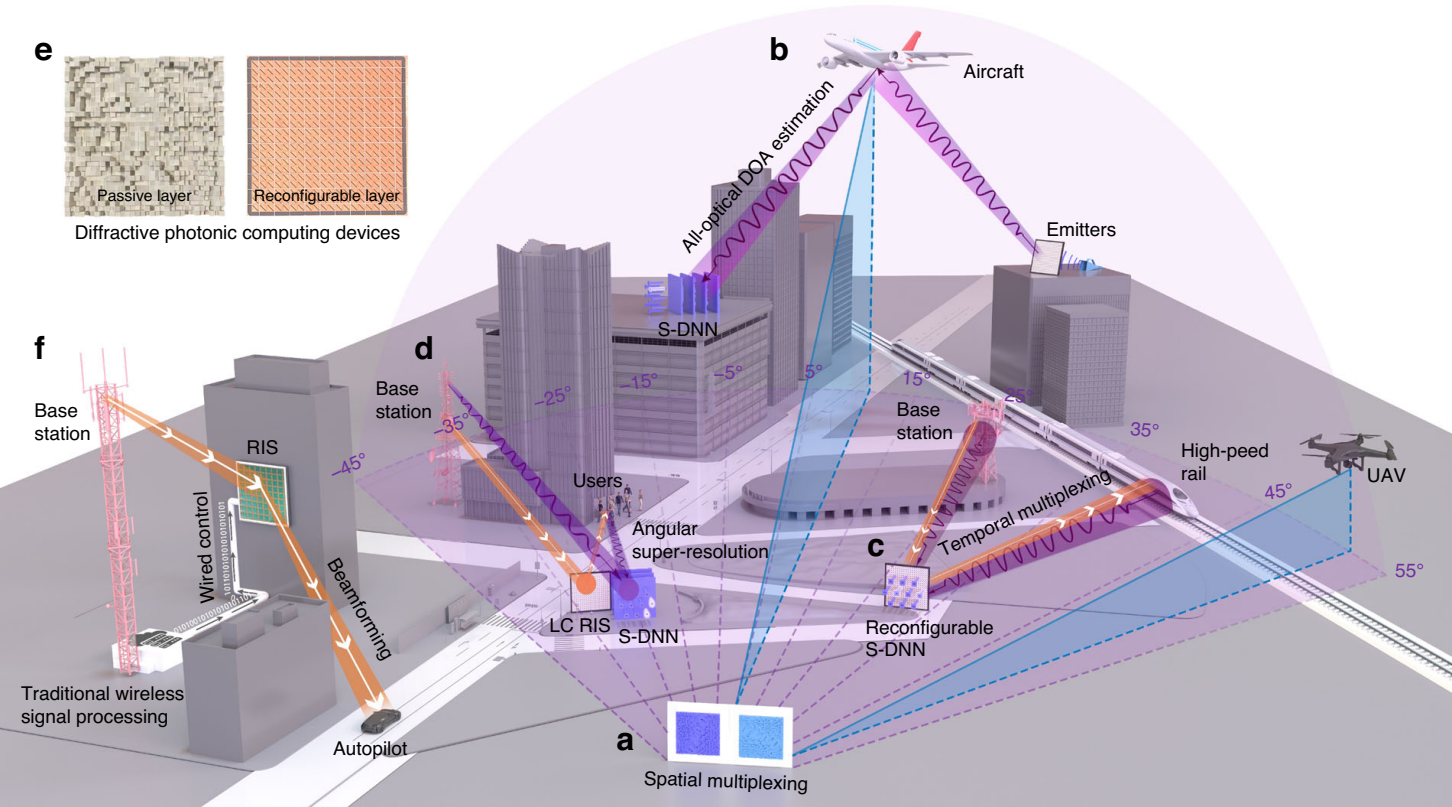
S-DNN for all-optical wireless sensing and communication. **a** S-DNNs can be spatially multiplexed for separately estimating the azimuth and elevation angular interval of targets over a wide field-of-view. **b** Four-layer S-DNN achieves DOA estimation with angular resolution beyond the diffraction limit, which can be applied for detecting and tracking targets with emitters. **c** Reconfigurable S-DNN utilizes LC RIS with temporal multiplexing to achieve DOA estimation for low-latency communication. **d** The three-layer S-DNN with super-resolution DOA estimation results are utilized to establish real-time RIS-based communication links between a base station and users. **e** Device pictures of the passive and reconfigurable diffractive layers utilized for integrated photonic computing, sensing, and communication. **f** The conventional RIS-based communication system relies on the base station to process wireless signals and send user locations, substantially increasing the communication latency

To address these challenges, we propose to construct super-resolution diffractive neural networks (S-DNN) for the all-optical DOA estimation over the broadband frequency range with angular resolution beyond the Rayleigh limit. Here, “all-optical” refers to using diffractive photonic computing devices to direct process signals carried by the EM wave. S-DNN can achieve DOA estimation at the speed of light with an angular resolution superior to the MUSIC algorithm without traditional radiofrequency circuits, ADCs, and digital signal processing. Different S-DNN models can be spatially or temporally multiplexed to flexibly estimate multi-target angles over the wide field-of-view (see Fig. 1). Specifically, S-DNN performs the 1D or 2D DOA estimation that separately or simultaneously estimates the elevation and azimuth angles (see Fig. S1). The input EM fields from different target sources are robustly classified into different angular intervals. For example, a single-layer S-DNN can estimate multi-target angles with a field-of-view of $100^{\circ}$ and an angular resolution of $10^{\circ}$ (see Fig. 1a). By increasing the diffractive modulation layer numbers, at any local field-of-view sizes of $30^{\circ}$ and $10^{\circ}$, the three-layer and four-layer S-DNNs achieve angular resolutions of $3^{\circ}$ and $1^{\circ}$, respectively, which exceeds the Rayleigh limit angular resolution of $4.37^{\circ}$ (see Fig. 1d, b). The applications of S-DNN for RIS-based communication systems using temporal or spatial multiplexing are illustrated in Fig. 1c, d, respectively. In Fig. 1b, the emitter scans and detects the aircraft, where the reflected EM waves are received by the S-DNN. In Fig. 1d, the estimated results of the S-DNN can be measured with power detectors and fed back to the field-programmable gate array (FPGA) in real time to further control the RIS to implement beamforming. The developed passive layers and liquid crystal (LC)-based RIS for diffractive photonic computing and communication are shown in Fig. 1e. For the conventional RIS-based communication system, the DOA estimation requires the base station to implement the pipeline of down-conversion, sampling, and digital signal processing, and the estimation results need to send to the RIS for establishing the communication links as shown in Fig. 1f and Fig. S1a (left). Differently, S-DNN empowers RIS-based communication systems with perception and edge computing capabilities, which facilitates low-latency beamforming tracking for real-time communications between base stations and high-speed mobile users with low power consumption.

## Results

### DOA estimation with S-DNNs beyond diffraction limits

The fundamental principle of S-DNN for DOA estimation is to classify the input EM field distribution of different target sources into different angular intervals (see Methods). S-DNN can be designed to work under 1D or 2D estimation mode for separately or simultaneously estimating the target elevation angle $\theta$ and azimuth angle $\varphi$. The architecture of S-DNN is constructed by cascading multiple diffractive modulation layers, followed by a detector array on the output plane (see Fig. S1b). Each detection region corresponds to an input angular interval, measuring the intensity of output EM fields. We implement the diffractive modulation layer with passive and reconfigurable intelligent surfaces, i.e., the PIS and RIS. Both PIS and RIS utilize sub-wavelength diffractive elements, i.e., the meta-atoms, to modulate the amplitude and phase of EM waves over broadband frequency ranges and generate large-scale optical interconnections between layers via diffractions (see Methods and Fig. S2). We designed the S-DNN to work at 5 G mmWave communication frequency band and experimentally validated with 1D estimation mode for separately estimating the elevation and azimuth angles. With accurate forward modeling, the parameters of each meta-atom, including the material thickness of PIS and control voltage of RIS, are optimized during the network training. The S-DNN learns to accumulate the energy of the incident plane wave from a target at a given angle to its corresponding detection region on the output plane. The target angular intervals are determined by finding the top-$K$ values of intensity measurement among detection regions, where $K$ represents the number of incident angular intervals having targets. The high degree-of-freedom design space with large-scale diffractive modulation enables S-DNN to generate super-oscillatory angular responses in different local angular ranges for super-resolution DOA estimations beyond the diffraction limit.

**Fig. 2:**
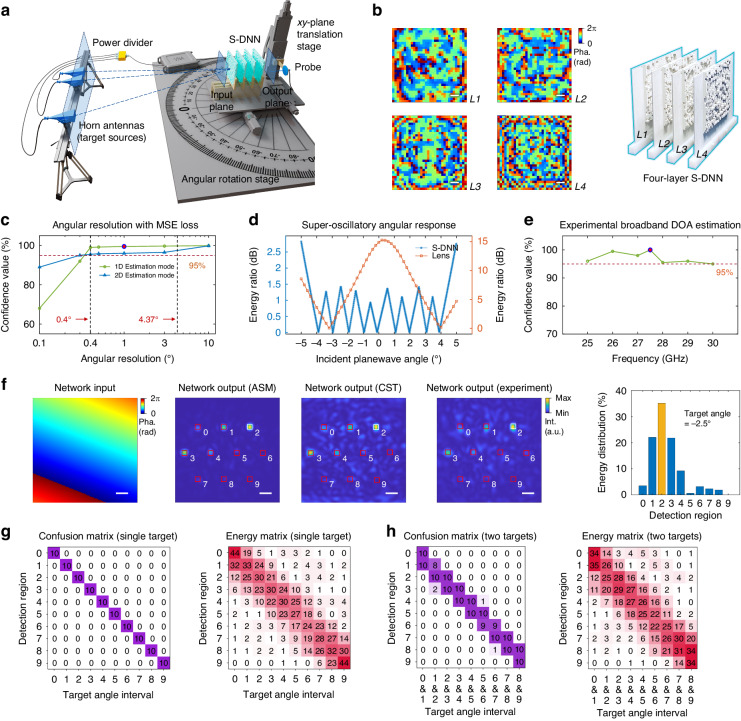
Super-resolution DOA estimation with four-layer S-DNN. **a** Schematic illustrating the experimental system. **b** Four-layer S-DNN after adaptive training and its implementation with PIS based on polytetrafluoroethylene (PTFE) RO3035 material for estimating elevation angle with $1^{\circ}$ angular resolution. **c**–**e** Characterizing four-layer S-DNNs, including the angular resolution under 1D and 2D estimation modes, the super-oscillatory angular response, and the confidence value at different frequencies for the model with $1^{\circ}$ angular resolution. **f** The exemplar inference results, evaluated with ASM, CST, and experiment, of a single input target with an elevation angle of $-2.5^{\circ}$ and an azimuth angle of $1^{\circ}$. **g**, **h** The experimental confusion matrices and energy distribution matrices were evaluated on the single-target and two-target testing datasets. The two targets are at adjacent angular intervals. Scale bar, 2 cm

We first demonstrate the multi-layer S-DNNs for the super-resolution DOA estimation at local angular ranges (see Fig. 2). As the elevation and azimuth directions are orthogonal in 3D space, the S-DNN models trained for elevation angle estimation can be used for azimuth angle estimation and verified with an azimuth angular rotation system after rotating the network with $90^{\circ}$ clockwise (see Fig. S3). The proposed experimental system for characterizing S-DNNs comprises a vector network analyzer (VNA) connected with horn antennas as target sources and a waveguide probe for detection, an azimuth angular rotation stage for carrying and rotating networks, and a *xy*-plane translation stage for setting the detection region of the waveguide probe (see Methods, Fig. 2a, and Fig. S4). We validate the proposed method by designing and fabricating a four-layer passive S-DNN based on PIS within a frequency range between 25 GHz and 30 GHz. To facilitate the experiments, the S-DNN is designed to perform the DOA estimation of elevation angles with $1^{\circ}$ angular resolution at the angular range of $[-5^{\circ} ,5^{\circ} ]$ (see Fig. 2b and Fig. S5). Moreover, different angular ranges with a field-of-view size of $10^{\circ}$, e.g., the angular range of $[45^{\circ} ,55^{\circ} ]$, can also be achieved by training different models (see Fig. S6). Each PIS has 32 × 32 modulation elements with element size setting to half of the central wavelength, i.e., 5.45 mm. The network parameters were re-trained with the dual adaptive training method (DAT)^39^ before fabrications to alleviate the model deviation (see Fig. 2b), and the positions of output detection regions were fine-tuned during the experiments. The DAT training process was supervised with the full-wave EM field simulation results that utilize the time-domain finite integration technology in CST Studio Suite.

The confidence value of four-layer S-DNN models for 1D and 2D DOA estimations, evaluated with the angular classification accuracy at different angular resolutions, are shown in Fig. 2c. The S-DNN was trained with the mean squared error (MSE) loss function for more robust estimation with higher energy percentage of correct categories. Besides, the models were evaluated on the boundary-free test datasets with 10,000 two-target test samples, where the angles in the angular interval boundary with a one-tenth of the angular interval range were not sampled. With a confidence value threshold of 95%, the model for both 1D and 2D DOA estimation modes can reach up to $0.4^{\circ}$ angular resolution that is over ten times higher than the diffraction-limited resolution defined by the Rayleigh criterion^40^. The angular resolution of the multi-layer S-DNN model can be further improved by increasing the network sizes, where the utilizing of the cross-entropy (CE) loss function during the training enables angular resolution 40–70 times higher than Rayleigh limits (see Fig. S7). For the complete angular sampling testing datasets that include angles at angular intervals, we developed the methods of flexible decision boundary and optoelectronic estimation to improve the model performance (see Supplementary Sections 8 and 9). The flexible decision boundary strategy compares the ratio of top-two power measurements to the pre-calibrated decision coefficients. The optoelectronic S-DNN architecture uses the least square method (LSM) to find the pre-calibrated prior angle that best matches the energy response of the unknown target.

In this work, we conduct the experimental evaluations of four-layer S-DNN with $1^{\circ}$ angular resolution. Figure 2d shows the comparison of angular response between S-DNN and a lens system under the same optical settings (see Fig. S8) by calculating the energy ratio of two detection regions with the largest and second-largest power values. The lens system has a smooth angular response that results in limited angular resolution. In contrast, S-DNN utilizes multi-layer subwavelength diffractive elements to effectively modulate the incident optical field and generate the super-oscillatory angular response at the angular range of $\left[-5^{\circ} ,5^{\circ} \right]$, which allows for the super-resolution DOA estimation. In addition to the frequency of 27.5 GHz, the experimental results demonstrate high confidence values above 95% over the broadband frequency range between 25 GHz and 30 GHz for a single input target (see Fig. 2e). The S-DNN models are numerically evaluated with angular spectrum method (ASM)^27^ on 10,000 test samples, which are further validated with CST and experimentally tested on 100 test samples. The exemplar DOA estimations of elevation angular interval for a single input target with an elevation angle of $-2.5^{\circ}$ and an azimuth angle of $1^{\circ}$ are shown in Fig. 2f. The results show the correctness of estimation as the second detection region corresponding to the angular interval of $[-3^{\circ} ,-2^{\circ} ]$ has the max detected intensity. The results also demonstrate the robustness of the model to achieve high similarity between the numerical and experimental results.

The confidence value of four-layer S-DNN models, evaluated with ASM, achieve 99.3% and 99.0% on the single-target and two-target test datasets, respectively (see Fig. S5). The corresponding angular estimation accuracies, evaluated with root mean square errors (RMSEs) that utilize the central angle of angular intervals as the ground truth, are $0.23^{\circ}$ and $0.24^{\circ}$, respectively; and the corresponding average energy percentages of the correct angular estimation are 34.6% and 29.8%, respectively. Each sample in the two-target test dataset includes two coherent targets distributed at the adjacent angular interval. During the experiment, the source signal from VNA is divided with a power divider and connected to two horn antennas spaced with $1^{\circ}$ that represent two target sources. The angular rotation stage rotates at a uniform step size to generate different angular test samples within the field-of-view. The corresponding experimental results of the confusion and energy distribution matrices, summarized over the test samples, are shown in Fig. 2g, h, validating high confidence values of four-layer all-optical S-DNN for DOA estimation with an angular resolution of $1^{\circ}$.

### Multiplexing S-DNNs with different configurations

**Fig. 3:**
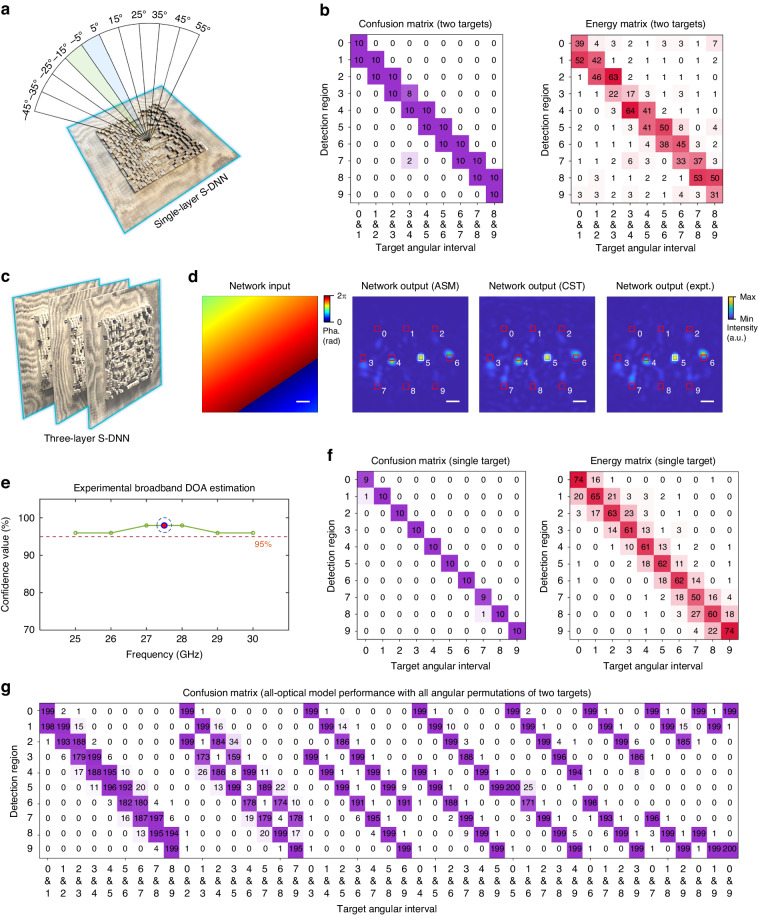
Multiplexing different S-DNNs. **a**, **b** Experimental results of a single-layer passive S-DNN, fabricated with PTFE F4BTME350 material, for the DOA estimation of two-target samples with an angular resolution of $10^{\circ}$ and an angular range of $[-45^{\circ} ,55^{\circ} ]$. **c**–**f** Experimental results of a three-layer passive S-DNN, fabricated with PTFE F4BTME350 material, for the broadband DOA estimation of single-target samples at a central frequency of 27.5 GHz and between the frequency range of 25 GHz and 30 GHz. The angular resolution is designed to be $3^{\circ}$ with an angular range of $[-15^{\circ} ,15^{\circ} ]$. **g** Confusion matrix of three-layer S-DNN, evaluated with ASM, for all-optical DOA estimation using the two-target test dataset with all angular permutations. Scale bar, 2 cm

S-DNNs can be spatially or temporally multiplexed to perform the coarse-to-fine DOA estimation, enabling the angular diffractive super-resolution over a wide field-of-view (see Fig. 1 and Fig. S1). In addition to the four-layer S-DNNs, we design different S-DNNs for the all-optical DOA estimation of single or multiple targets with the angular resolution of $15^{\circ}$, $10^{\circ}$, $3^{\circ}$, and $1^{\circ}$, corresponding to the field-of-view size of $150^{\circ}$, $100^{\circ}$, $30^{\circ}$, and $10^{\circ}$, respectively, at given angular ranges. Multiplexing S-DNNs, configured with different angular resolutions and angular ranges, allows us to design the system to achieve the super-resolution DOA estimation over a wide field-of-view. For instance, to achieve the DOA estimation at an angular range of $\left[-45^{\circ} ,55^{\circ}\,\right]$ with an angular resolution of $1^{\circ}$, the single-layer S-DNN model with an angular resolution of $10^{\circ}$ can first be utilized to cover the field-of-view (see Fig. 3a). Then, the four-layer S-DNN models can be utilized to achieve $1^{\circ}$ angular resolution at each $10^{\circ}$ angular interval of $\left[-45^{\circ}\,+10i,-35^{\circ}\,+10i\right]$ with $i=0,\ldots ,9$.

The numerical and experimental results of a single-layer S-DNN for estimating target elevation angles with a range of $\left[-45^{\circ} ,55^{\circ} \right]$ and a resolution of $10^{\circ}$ are shown in Fig. 3 and Figs. S9 and S10. The confidence values of the model, evaluated with ASM at the central frequency of 27.5 GHz, on the single-target and two-target test datasets with 10,000 samples are 98.7% and 98.0%, respectively. Similar to four-layer S-DNN, the experimental results of confusion and energy distribution matrices, summarized on 100 single-target and 100 two-target test samples, show the high confidence values and average energy percentages of correct angular categories. The broadband DOA estimation of a single target also demonstrates the high confidence values of the model above 95% between 25 GHz and 30 GHz. Besides, the single-layer S-DNN can also achieve a field-of-view of $150^{\circ}$ and $30^{\circ}$ for single-target test samples, corresponding to the angular resolution of $15^{\circ}$ and $3^{\circ}$, respectively (see Figs. S11 and S12). Besides, the single-layer S-DNN with $4^{\circ}$ angular resolution can achieve the super-resolution DOA estimation for two target sources from arbitrary angular intervals (see Fig. S13).

**Fig. 4:**
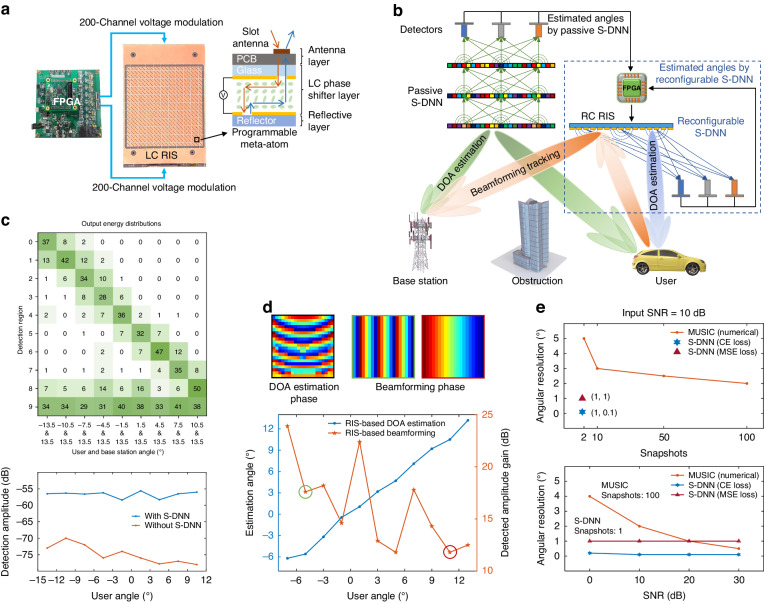
S-DNN for integrated sensing and communication. **a** The reflective LC RIS is controlled by 400-channel voltages through FPGA. The voltage applied to the liquid crystal layer changes its dielectric constant for modulating the phase of incident EM fields. **b** Schematic illustrating the application of DOA estimation with passive and reconfigurable S-DNNs for RIS-based communications. **c** Experimental output energy distribution of the three-layer passive S-DNN for angular estimation of users and base stations (top). Based on the results of the S-DNN, RIS accurately steers the base station beam to the user, improving the receiving gain (bottom). **d** RIS-based communication systems using the angular estimation result of reconfigurable S-DNNs substantially improve the detected signal strength. **e**, S-DNN advanced over the conventional DOA estimation with MUSIC in terms of the snapshots and angular resolution. The S-DNN is more robust and achieves higher angular resolution than MUSIC at low input SNR

To improve the model confidence value for multi-target samples, we designed and constructed a three-layer S-DNN for the super-resolution angular estimation with $3^{\circ}$ resolution at the angular range of $\left[-15^{\circ} ,15^{\circ} \right]$ (see Fig. 3c). The three-layer S-DNN model is evaluated with different two-target testing datasets, including datasets with two targets at an adjacent angular interval (see Fig. S14d), separated by one angular interval (see Fig. S14f), and with all angular permutations of two targets (see Fig. 3g and Fig. S15a), achieving the confidence values of 95.7%, 99.5%, and 94.9%, respectively. The corresponding angular estimation accuracies, evaluated with RMSEs, are $0.81^{\circ}$, $0.77^{\circ}$, and $0.88^{\circ}$, respectively; and the corresponding average energy percentages of the correct two-target angular estimation are 40.6%, 34.9%, and 33.7%, respectively. Figure 3d demonstrates the robustness of the model to achieve high similarity between the numerical and experimental network outputs, which correctly estimates the elevation angular interval of $[0^{\circ} ,3^{\circ}\,]$ for an exemplar single input target with an elevation angle of $1.5^{\circ}$ and an azimuth angle of $1^{\circ}$. The 3D EM field dynamics of the network are shown in Supplementary Videos 1 and 2. The experimental results in Fig. 3e further validates the high confidence values above 95% of the three-layer S-DNN for the broadband DOA estimation between the frequency range of 25 and 30 GHz. Figure 3f shows the experimental confusion and energy distribution matrices of 100 single-target test samples at a central frequency of 27.5 GHz, and the experimental results of the two-target test samples are shown in Fig. 4c. For the complete angular sampling testing datasets, the flexible decision boundary (see Fig. S16) can be utilized for improving the model performance for single-target samples, and the optoelectronic architecture (see Fig. S17) can be utilized for improving the model performance for both single-target and multi-target samples. The optoelectronic DOA estimation improves the model confidence value of the three-layer S-DNN from 94.9% to 99.5% on the two-target test dataset with all angular permutations and complete angular sampling (see Fig. S17c).

### S-DNN for integrated sensing and communication

Based on the edge computing and broadband angular perceptron capability of S-DNNs, we demonstrate the application of S-DNN for RIS-based mmWave communication to achieve low-latency integrated sensing and communications. With the support of passive or reconfigurable S-DNNs as the all-optical edge computing devices to achieve the super-resolution DOA estimation, RIS can autonomously sense the EM environment independent of base stations, enabling a real-time communication link between the base station and high-speed mobile users (see Fig. 4). In this work, we develop the reflective LC RIS system, which comprises 20 × 20 programmable meta-atom to modulate the phase of incident EM field for beamforming communication and implement the reconfigurable S-DNN (see Fig. 4a and Methods). Each meta-atom element has a phase modulation accuracy of 5 bits controlled by the voltage from the field programmable gate array (FPGA). For the phase distribution to be loaded on the RIS, the corresponding supply voltage is applied to each cell of the liquid crystal layer, where the response time to switch the refractive index of liquid crystal to the target value requires less than 500 ms.

The system schematic and pipeline of fusing all-optical edge computing capability of passive S-DNN for the RIS-based communication system are depicted in Fig. 4b. The passive S-DNN performs the all-optical angle estimation of multiple targets at extremely low latency, depending on the detection speed, after receiving EM waves from the base station and users. Based on the estimation results of S-DNN, the FPGA optimizes the beamforming phase and configures RIS to reflect the EM wave from the base station to the user to realize beamforming tracking^32^, which bypasses obstacles to establish real-time communication links. The simultaneous DOA estimation of the base station and the user is demonstrated by utilizing the three-layer passive S-DNN in Fig. 3c. During the experiment, two horn antennas are utilized to represent the base station and user, respectively (see Fig. S4c, top). The incident angle of the base station is fixed at 13.5°, and the incident angle of the user changes from −13.5° to 10.5° at a step size of 3°. From the output energy distribution of ten detection regions in Fig. 4c, S-DNN achieves super-resolution DOA estimation for the base station and the user. With the output of passive S-DNN, RIS can optimize the beamforming phase and establish a communication link between the base station and the user, realizing an average detected amplitude gain of 17.9 dB (see Fig. 4c, bottom). Without S-DNN, the RIS cannot precisely steer the beam, so the user antenna can only detect the ambient noise.

The reconfigurable S-DNN utilizes time-division multiplexing for both DOA estimation and beamforming, based on the programmability and high modulation accuracy of stand-alone RIS (see Fig. 4b). The trained phase modulation layer for DOA estimation and the beamforming phase for the user angle at −5° and 11°, under the known base station angle of −13.5°, are shown in Fig. 4d, top-left and top-right, respectively. We utilize the optoelectronic architecture to improve the angular estimation accuracy at the user angular range of [−15°, 15°], which achieves an RMSE of 0.19° (see Fig. S18). To facilitate the beamforming, we further evaluate the DOA estimation and beamforming communication performances by placing the user at the angular range of [−7°, 13.5°] (see Fig. 4d, bottom). The reconfigurable SDNN achieves the high-precision angle estimation with an RMSE of $0.44^{\circ}$. Based on the angles of the base station and different users, as detailed in Supplementary Section 10, the reconfigurable S-DNN optimizes beamforming phases and converts it to 400-channel voltages to configure the modulation elements, realizing an average detected amplitude gain of 16.1 dB at the user antenna. Besides, as illustrated in Fig. S19, with the advantage of reconfigurable layers, the source number estimation can also be conducted to provide additional prior information to S-DNN for a multi-source super-resolution DOA estimation task. Figure 4e further illustrates the advantages of S-DNN for DOA estimation over the conventional method using MUSIC, especially under low snapshots and input SNR (see Supplementary Section 12). With the same number of modulation elements, S-DNN only requires a single snapshot and can achieve higher angular resolution with more robust estimation results to input noise than MUSIC, facilitating RIS-based communication with low latency.

## Discussion

The network size of S-DNN can easily be scaled up to have more meta-atoms, diffractive layers, and detection regions. Compared with the single-layer model, multi-layer S-DNN has a higher degree of freedom and larger network scale to perform the multi-level diffractive modulation of the input EM field, which achieves more accurate DOA estimation for multiple targets. Besides, the multi-layer S-DNN has the capability for the DOA estimation of more targets distributed at different angular intervals. More meta-atoms at each layer correspond to a larger perception aperture for facilitating higher angular resolution (see Fig. S7). Furthermore, the number of detection regions at the S-DNN output plane can be increased from 10 to 20, thus increasing the field-of-view while maintaining angular resolution (see Fig. S20). Three five-layer S-DNNs with 10, 15, and 20 detectors, realizing $3^{\circ}$ resolution with a field-of-view size of 30°, $45^{\circ}$, and $60^{\circ}$, were evaluated with two-source test datasets and can achieve high confidence values of 99%, 97%, and 90%. As shown in Fig. S20a, S-DNN is optimized to move the super-oscillatory angular frequency regions into the frequency region so that the detectors can capture the angular frequency higher than the diffraction limit. Nonetheless, the increased field-of-view will increase the size of super-oscillatory angular frequency regions, making the detection more challenging.

Based on the dispersion resistance of S-DNNs with broadband training, S-DNN is capable of accurately estimating DOA with a high confidence value above 95% with a 9 GHz maximum bandwidth from 23 to 32 GHz (see Fig. S15). We further analyze the bandwidth of S-DNNs under different target numbers (see Fig. S21). As the number of sources increases from 1 to 5, it becomes more challenging for S-DNN to achieve multi-source DOA estimation, which results in reduced bandwidth. By increasing the layer number of meta-structures, the performance of S-DNN can be substantially improved to achieve more target source estimation and wider bandwidth. In this work, we implement the S-DNN models with 1D DOA estimation that separately estimate the elevation and azimuth angles. The experimental system can be upgraded to a 2D rotation stage to characterize S-DNNs with 2D DOA estimation. Higher angular resolutions in Fig. 2c and Fig. S7 can be approached during the experiments by utilizing the in-situ training methods^39,41^ for training reconfigurable S-DNNs to adapt the model to systematic errors.

The detection channels of VNA can be scaled up to ten channels to have an upper bound estimation latency of 67 ns. Then, the computing speed of the constructed four-layer passive S-DNN, each layer with 32 × 32 meta-atoms, is 6.94 TOPS, which increases to 3.78 POPS by increasing the layer number to five with 512 $\times$ 512 meta-atoms at each layer (see Supplementary Section 13). The passive layer of S-DNN does not require the power supply, and the energy consumption of each reconfigurable LC RIS panel with 20 $\times$ 20 elements is ~0.5 W. To facilitate the implementation, the radio frequency switch, e.g., TLSP10T26.5G40GA (Talent Microwave Inc.), can be utilized to achieve fast switching of the ten detection regions, where the switching speed is 100 ns and the detection speed is 67 ns, for total response speed of 1.67 µs. Notice that the state-of-the-art multi-channel radio direction-finding device Rohde & Schwarz DDF5GTS includes ~9 antennas to implement the MUSIC algorithm with an angular resolution of less than $20^{\circ}$, which has the estimation latency of 1 ms and the power consumption of 650 W. S-DNN only requires a single snapshot to achieve an estimation latency of 1.67 µs and 67 ns with the radio frequency switch and ten-channel VNA, respectively, improving the estimation latency for more than two and four orders of magnitude, respectively. Future works aim to develop a metasurface power detector^42^ as a cost-effective alternative to the expensive VNA, enabling precise measurement of the field distribution at the S-DNN output plane as well as enhancing system integration. Besides, due to the mixing of different target signals into one channel for sampling, the MUSIC algorithm fails to process coherent targets. In contrast, S-DNN directly processes the EM field from target sources to map the EM waves from different angles to corresponding detection regions over the broadband frequency ranges, allowing it to process multiple coherent or incoherent sources. Besides, previous RIS-based angle estimation methods^38,43,44^ failed for multi-target estimation and consumed massive electronic computing resources; the S-DNN addresses the challenge of positioning multiple mobile users and has substantially improved energy efficiency.

DOA estimation at the speed of light makes it ideal for autopilot and high-speed rail communications, as illustrated in Fig. 1. Equipped with a high-power emitter, S-DNN can be applied to radar target detection and tracking, as well as satellite navigation and positioning. Additionally, S-DNN can use reconfigurable transmissive metasurfaces^28^ to continuously switch the phase distribution to achieve 1° angular resolution in the angular range of $\left[-45^{\circ} ,55^{\circ} \right]$. Reconfigurable S-DNN can also be applied to different tasks by training and deploying different models, such as object recognition^45^, holographic imaging^46^, varifocal meta-devices^47^, encrypted information transmission^48^, pupil phase retrieval^49^, and broadband application^50,51^. Different tasks can also be multiplexed into different wavelengths in parallel with the wavelength-division multiplexing^52^ and polarization multiplexing^53^. In summary, we have presented a diffractive photonic computing paradigm to directly process EM waves for all-optical DOA estimation. The proposed approach enables integrated in-memory sensing and computing with low latency and power consumption that facilitates the application in intelligent wireless communication networks.

## Materials and methods

### The principle of S-DNN for DOA estimation

The principle of super-resolution diffractive neural network, i.e., S-DNN, for DOA estimation with 1D or 2D mode is demonstrated in Fig. S1b. The S-DNN takes the electromagnetic (EM) field distribution of incident waves generated from a target signal source at the far-field plane as the input to recognize its belonging angular interval. Let the input plane center of S-DNN as the coordinate origin, then the EM field distribution of a target source at the ${z}_{0}$ axial plane with an elevation angle of $\theta$ and an azimuth angle of $\varphi$ can be approximated as the far-field plane wave:

1 $$E\left(x,\,y,\lambda \right)={A}^{{\prime} }\exp \left\{{jk}(x\sin \theta +y\cos \theta \sin \varphi )\right\}+{n}_{\rm{noise}}$$

where ${A}^{{\prime} }=A\exp ({jk}{z}_{0}\cos \theta \cos \varphi )$ is the constant complex value with $A$, $k=2\pi /\lambda$, and $\lambda \in \left[{\lambda }_{1},{\lambda }_{2}\right]$ being the amplitude, vacuum wavenumber, and working wavelength, respectively; ${n}_{\rm{noise}}$ denotes the spatial random Gaussian noise. The far-field distance ($z={z}_{0}$) is set to be larger than the Rayleigh distance for generating planar optical wavefront, where different ${z}_{0}$ causes the uniform phase delay of the wavefront that doesn’t affect the estimation results of the designed S-DNN in this work. Equation (1) shows that different target sources with different elevation angle $\theta$ and azimuth angle $\varphi$ generate different phase patterns at the input plane of S-DNN.

S-DNN classifies the input phase patterns of different target sources into different angular intervals, which estimates the elevation and azimuth angles separately under 1D estimation mode and simultaneously under 2D estimation mode. For the multiple input targets, the input field is the superposition of multiple plane waves with different incident angles, and S-DNN can map the energy of each plane wave to the detection region corresponding to the respective incident angle. Therefore, the angles of incident plane waves can be determined by finding the top-$K$ values of intensity measurements among detection regions for $K$ incident angles. We set $K=1,\ldots ,10$, representing that $K$ different incident angular intervals have targets. The proposed method utilizes the diffractive super-resolution characteristic of passive or reconfigurable S-DNN with spatial or temporal multiplexing to perform the coarse-to-fine angular estimation for the wide field-of-view and high-resolution DOA estimation.

### The network configurations of S-DNN

The S-DNN is designed to work at a 5 G mmWave communication frequency band with the wavelength range from ${\lambda }_{1}$ to ${\lambda }_{2}$ and the central wavelength of ${\lambda }_{0}$. In this design, the modulation element size is set to approximate ${\lambda }_{0}$/2 for both passive and reconfigurable implementations. We specify ten detection regions on the output plane, each corresponding to an input angular interval, measuring the intensity of output EM fields and performing the nonlinearity to obtain the DOA estimation results. The size of each detection region is set to be 5${\lambda }_{0}$/8* 5${\lambda }_{0}$/8 to match the size of the waveguide probe, which is used to detect EM fields. Ten waveguide probes are placed in ten detection regions with a separation distance exceeding four wavelengths, where the mutual coupling effect can be negligible. In this work, the frequency range was set between 25 and 30 GHz for passive S-DNNs and between 25 and 27.5 GHz for reconfigurable S-DNNs.

To improve the accuracy of the numerical model, for each diffractive modulation layer with a modulation element number of *N* × *N* and a modulation element size of *M* × *M*, the grid size was set to be *M*/4 × *M*/4 with a grid number of 4*N* × 4*N*. We set *N* = 32 and *M* = ${\lambda }_{0}/2$ for passive S-DNNs in the experiment, corresponding to an aperture size $D$ = 16${\lambda }_{0}$. Moreover, each PIS had a substrate thickness of 3 mm and was added to the surrounding frame with a width of 50 mm to facilitate the support and alignment, resulting in a size of 274.54 mm × 274.54 mm. The diffractive layer distance and the output plane to the last layer distance were set to 5${\lambda }_{0}$ to enable the fully connected neural network structure. Besides, to narrow the search space and reduce the variation of the adjacent elements of the phase modulation layer, the sigmoid function was used to constrain the material thickness to 0–*H* with $H={\lambda }_{0}$ and phase modulation values to 0–2*π* for the passive and reconfigurable S-DNNs, respectively.

### The design and fabrication details of PIS and RIS

For the passive S-DNNs, since the central working frequency is 27.5 GHz, corresponding to the central wavelength ${\lambda }_{0}$ = 10.9 mm, the modulation element size of PIS was set to be 5.45 mm. After evaluating the passive S-DNN with CST Studio Suite (Dassault Systèmes Simulia Corp.) with open space boundary conditions, the 3D models are exported for fabrication. PIS is made by mixing polytetrafluoroethylene F4B (PTFE-F4B) material with uniform nano-ceramics and glass fiber cloth. This material has superior spatial isotropic properties and has a stable dielectric constant $\varepsilon$ with minimal loss when used at frequencies below 40 GHz. In this work, we utilize three types of PTFE materials, including F4BTME350, F4BTMS350, and RO3035, with the dielectric constant of $4.03+0.04i$, $3.65+0.04i$, and $3.89+0.016i$, respectively. F4BTME350 is the PTFE glass fiber cloth nano-ceramic copper clad laminate, and F4BTMS350 is the PTFE superfine glass fiber cloth ceramic-filled substrate. Both F4BTME350 and F4BTMS350 materials are manufactured by Wangling Company in Taizhou, China. The RO3035 material is produced by Elec & Eltek and is prepared by laminating 0.5 mm thick RO3035 material (PTFE ceramic material) and 0.1 mm RO4450F material (PTFE ceramic fiberglass cloth semi-cured sheet) from Rogers Corporation. The F4B material layer is fabricated with the precision computer numerical control (CNC) machine tools to form diffractive elements. Since the CNC machine tool has an axial machining precision of ~0.1 mm, the phase modulation bit depth of PIS is ~7-bit.

For the construction of reconfigurable S-DNNs, the developed liquid crystal-based RIS system has 20 $\times$ 20 effective programmable meta-atom elements, where the 400-channel modulation voltages are programmed with FPGA. Each element of LC RIS has a 5-bit phase modulation precision with a size of 5.5 mm × 5.5 mm, comprising an antenna layer, an LC phase shifter layer, and a reflective layer. The modulation voltage changes the dielectric constant of the LC phase shifter layer and modulates the phase of incident EM fields. The LC RIS works under the reflection mode (see Fig. S2b), which can be programmed to perform beamforming communication or switch between different models for the DOA estimation at different angular ranges.

### The training details of S-DNN

The forward EM field propagation of S-DNN is modeled over a broadband wavelength range, where the models of passive and reconfigurable diffractive modulation layers are detailed in Supplementary Sections 1 and 2. The Rayleigh-Sommerfeld diffraction, implemented with the angular spectral method (ASM), was utilized to model the broadband EM field propagation between layers^52^. The angular spectrum method is written in Python and developed on top of the popular machine-learning library, Pytorch. The zero padding was included at the periphery of diffractive layers to ensure the boundary condition of the numerical model. The outputs of S-DNN are measured with detectors and compared with the ground truth targets of the DOA estimation task to define the loss function. During the training, the network coefficients are optimized with the error backpropagation method to minimize the loss function. We utilized the mean square error (MSE) loss to facilitate more robust models for physical experiments and cross-entropy (CE) loss to demonstrate its potential angular resolution upper bound. The learning rate, batch size, and epoch number were set to 0.01, 128, and 100, respectively. Notice that the broadband modulation model of PIS in Supplementary Section 1 is an approximate model with respect to the EM field modeling for facilitating the effective training of passive S-DNN. Thus, for the four-layer S-DNN, we further utilize the dual adaptive training method (DAT)^39^ under the supervision of full-wave EM field simulation results that are obtained from the time-domain finite integration technology in CST. The DAT is utilized to fine-tune the material thickness of each diffractive element during the training so that the designed passive S-DNN can adapt to the model deviation.

### Experimental system

All the experimental results of S-DNN were obtained by measuring the magnitude of the S21 parameter using the Keysight P5006B vector network analyzer (VNA) in a microwave anechoic chamber. To obtain the DOA estimation results of S-DNN, the VNA generated an mmWave signal at Port 1 and connected it to two horizontally polarized antennas as sources through a power divider. A waveguide probe as detector was connected to Port 2 of the VNA via cables to measure the magnitude of the S21 parameter to obtain the intensity of network output detection regions. The distance between the horizontally polarized antennas and the S-DNN was 5.5 m to ensure the far-field condition, so the incident wavefront of the S-DNN was approximated as a plane wave. The two antennas were placed on the sliding guide rail to adjust the position and spacing, respectively. The waveguide probe was fixed on the XY mechanical platform to scan the output energy distribution of S-DNN. The XY mechanical platform was driven by two vertically placed stepper motors, which precisely controlled the movement of the waveguide probe in both horizontal and vertical directions within a range of 65 cm with 0.01 mm accuracy. The scanning position of the waveguide probe was determined by ten detection regions, and the scanning step size was ${\lambda }_{0}/8$. The S-DNN was fixed on the angular rotation stage with the rotation axis located at the center of the S-DNN. The angular rotation stage was driven by a stepper motor to rotate within the range of $[0^{\circ} ,360^{\circ} ]$ in the azimuth direction with a rotation accuracy of $0.01^{\circ}$, so as to precisely control the angle of the incident plane wave. The customized scanning program of the angular rotation stage and XY mechanical platform communicated with the VNA to perform the measurements, and the output energy distributions corresponding to different incident angles were obtained.

In the case of the reconfigurable S-DNN measurements, we placed the waveguide probe obliquely in front of the RIS with a distance of $15{\lambda }_{0}$ to avoid occlusion between the waveguide probe and the incident wave. Since the RIS had a $45^{\circ}$ linear polarization, the waveguide probe and the transmitting antenna were rotated $45^{\circ}$ with a custom-made adapter plate. During the beamforming measurement with RIS, the two $45^{\circ}$ linear polarization antennas were connected to the two ports of the VNA as source and detector, respectively. The S-DNN and RIS shared an identical coordinate system and were positioned adjacently, ensuring consistency in the incident angle. To minimize multipath propagation and reflection, the experimental environment (except the S-DNN) was covered with microwave absorbing material.

### Generating training and testing datasets for S-DNN

The training and testing datasets of S-DNN for DOA estimation were obtained by generating the far-field plane waves from different target sources with the elevation angle of $\theta$ and the azimuth angle of $\varphi$, and setting with random ${z}_{0}$ for random initial phase value. In this work, both training and testing datasets have 10,000 samples for each DOA estimation task. We include the spatial random Gaussian noise ${n}_{\rm{noise}}$ to the input fields and set the signal-to-noise ratio (SNR) to 10 dB during the training and testing. For example, in the first stage of the wide field-of-view DOA estimation task in Supplementary Fig. 1b, we train S-DNN to estimate the azimuth angular interval by setting the field-of-view to $100^{\circ}$ with an angular range $\varphi \in \left[-45^{\circ} ,55^{\circ} \right]$. The angular range was divided into ten intervals $\left\{{\varphi }_{i},i=0,\ldots ,9\right\}$, each with $10^{\circ}$ angular range ${\varphi }_{i}\in \left[-45^{\circ}\,+10i,-35^{\circ}\,+10i\right]$, corresponding to ten detection regions with ground truth labels of No. $i$ ($i=0,\ldots ,9$) on the output plane, respectively. For each 10° angular interval of ${\varphi }_{i}$, we generate 1,000 phase distributions according to Eq. (1) by randomizing azimuth angles: ${\varphi }_{{ij}}=-45^{\circ} +10i+(-35^{\circ}\,+10i-(-45^{\circ}\,+10i))\,\cdot\,{x}_{j}$, where ${x}_{j}$ denotes a random value between 0 and 1 with $j=1,\ldots ,1000$. Furthermore, we set the random value of the elevation angle $\theta$ within the same angular range of $\left[-45^{\circ} ,55^{\circ} \right]$ for each phase distribution, which enables the azimuth angular interval estimation with S-DNN that is robust to the elevation angle variation. Therefore, there are in total 10,000 training samples, each corresponding to an azimuth angle of ${\varphi }_{{ij}}$ with a ground truth label of $i$. Besides, to improve the model performance for multiple input targets, we further generate the multi-target training samples in addition to the single-target training samples, where the EM field of each multi-target sample is obtained by superimposing the EM field of the single-target samples. The testing dataset is generated in the same way. To facilitate the model evaluation and experiment, the angles around the angular interval boundary with a range of one-tenth of each angular interval were not sampled in the boundary-free testing datasets.

The same training and testing dataset generation method was utilized for other S-DNN models with different field-of-views and range of angular intervals. In the second stage of the super-resolution DOA estimation task at local azimuth angular regions in Supplementary Fig. 1b, each $1^{\circ}$ angular range of ten intervals of the S-DNN model with a field-of-view of $10^{\circ}$ and ${\varphi }^{{\prime} }\in \left[-5^{\circ} ,5^{\circ} \right]$ can be formulated as: ${\varphi }_{i}^{{\prime} }\in \left[-5^{\circ} +i,-4^{\circ} +i\right]$, $i=0,\ldots ,9$. Therefore, the training and testing samples can be generated as: ${\varphi }_{{ij}}^{{\prime} }=-5^{\circ}\,+i+(-4^{\circ}\,+i-(-5^{\circ}\,+i))\,\cdot\,{x}_{j}$. We also include the random value of the elevation angle ${\theta^{\prime}}$ within the same angular range $\left[-5^{\circ} ,5^{\circ} \right]$ to enable the robustness of estimation of the elevation angle. With the generated training and testing datasets, the S-DNN learns to perform the DOA estimation task by mapping the incident plane waves from single or multiple target sources to the detection regions on the output plane. The target at the $i$-th angular interval is mapping to the No. $i$ detection region.

### Supplementary information

Supplementary Information

Supplementary Video 1

Supplementary Video 2
