# Supplementary material for: Super-resolution diffractive neural network for all-optical direction of arrival estimation beyond diffraction limits
Source: Light Sci Appl. 2024 Jul 10;13:161. doi: 10.1038/s41377-024-01511-4 (PMC11237115; doi:10.1038/s41377-024-01511-4)
Supplement: Supplementary file 1 — Supplementary Information [file 41377_2024_1511_MOESM1_ESM.docx]

Supplementary Information for

**Super-resolution diffractive neural network for all-optical direction of arrival estimation beyond diffraction limits**

Sheng Gao^1^*, Hang Chen^1^*, Yichen Wang^1^, Zhengyang Duan^1^, Haiou Zhang^1^,

Zhi Sun^1^, Yuan Shen^1,2^, and Xing Lin^1,2^†

^1^Department of Electronic Engineering, Tsinghua University, Beijing, 100084, China

^2^Beijing National Research Center for Information Science and Technology, Tsinghua University, Beijing, 100084, China

*These authors contributed equally to this work.

†Corresponding author. E-mail: lin-x@tsinghua.edu.cn

**This file includes:**

Supplementary Materials

Supplementary Figs. S1 to S21

References

**Other Supplementary Materials for this manuscript include the following:**

Supplementary Videos 1 to 2

1. **The broadband modulation model of passive intelligent surface**

Passive S-DNN is constructed with a single or multiple passive intelligent surfaces (PIS) and works under the transmission mode (see Fig. S2a). Besides the substrate, each diffractive element has a material thickness of $h$ that will be optimized between 0 and $H$ during the training, following an air thickness of $H-h$. Considering broadband S-DNN working in a wide wavelength range from $\lambda_{1}$ to $\lambda_{2}$ with the central wavelength of $\lambda_{0}$, the maximum height is constrained to $H=\left( \lambda_{1}+\lambda_{2} \right)/2=\lambda_{0}$. The passive diffractive element generates complex transmission coefficients to modulate the amplitude and phase of the incident EM wavefront. By considering the inter-reflection of the EM field at each diffractive element during the optimization process and ignoring the crosstalk between adjacent diffractive elements under the smooth constrain, the complex transmission coefficient of a diffractive element can be expressed as [s1]:

$$\left\{ \begin{aligned} t_{m}\left( h,\lambda\right)=\frac{t_{12}t_{23}exp(j2\pi nh/\lambda)}{1+r_{12}r_{23}exp(j4\pi nh/\lambda)}\text{ } \\ t_{a}\left( H-h,\lambda\right)=\exp\left( j2\pi\left( H-h \right)/\lambda\right)\text{ } \\ t\left( h,\lambda\right)=t_{m}\left( h,\lambda\right)t_{a}\left( H-h,\lambda\right)\text{ } \end{aligned} \right.\text{ }\text{ }\text{(1)}$$

where $t_{m}(h,\lambda)$ and $t_{a}(h,\lambda)$ are the transmission coefficients of material and air with $\lambda\in\left[ \lambda_{1},\lambda_{2} \right]$; $t_{12}=2/{(1+n)}$, $t_{23}={2n}/{(1+n)}$, $r_{12}={(1-n)}/{(1+n)}$, and $r_{23}=-r_{12}$ with $n\in\left[ n_{1},n_{2} \right]$ being the corresponding refractive index of the material.

1. **The broadband modulation model of reconfigurable intelligent surface**

Reconfigurable S-DNN is constructed with reconfigurable intelligent surfaces (RIS) and works under the reflection mode (see Fig. S2b), which can be programmed to switch between different models for the high-resolution DOA estimation at different angular ranges. The mmWave liquid crystal RIS achieves the effective phase modulation of EM fields by integrating the antenna layer, liquid crystal phase shifter layer, and reflective layer. As the liquid crystal is an anisotropic material, the deflection direction $\theta$ can be adjusted to achieve reconfigurability by controlling the voltage $V$ between the electrodes of the liquid crystal phase shifter layer, which can be formulated as [s2]:

$$\frac{V}{2V_{th}}=\frac{1}{\pi}\int_{0}^{\theta} \left( \frac{\cos^{2} \alpha+k\sin^{2} \alpha}{\sin^{2} \theta-\sin^{2} \alpha} \right)^{\frac{1}{2}}d\alpha\text{ }\text{ }\text{ (2)}$$

where $V_{th}$ is the threshold voltage for liquid crystal deflection, $k$ is the elastic constant. The deflection direction $\theta$ of the liquid crystal affects its effective dielectric constant $n\left( \theta\right)$:

$$n\left( \theta\right)=\left( \frac{\cos^{2} \theta}{n_{\perp}^{2}}+\frac{\sin^{2} \theta}{n_{\parallel}^{2}} \right)^{-\frac{1}{2}}\text{ }\text{ }\text{ }\text{ }\text{(3)}$$

where $n_{\perp}$ and $n_{\parallel}$ represent the dielectric constants when the liquid crystal turns to the horizontal and vertical directions, respectively. Therefore, the phase delay $\varphi$ of the EM wave modulated by the liquid crystal phase shifter can be formulated as:

$$\varphi(\theta,\lambda)=\frac{2\pi}{\lambda}\left( n\left( \theta\right)-n_{\perp} \right)d\text{ }\text{ }\text{ (4)}$$

where $d$ denotes the thickness of liquid crystal phase shifter layer. Eqs. (2), (3), and (4) shows that applying different control voltages continuously change the dielectric constant of liquid crystal and achieves the high-precision phase modulation.

Different from the passive S-DNN, the detection plane of reconfigurable S-DNN working under the reflection mode is on the same side of the incident plane wave. To avoid the occlusion between detector array and incident waves, the detection plane is set to the oblique front instead of central front during the design, where the virtual detection plane is also mirrored obliquely behind the reconfigurable S-DNN, as shown in Fig. S2b, with the constructed experimental system demonstrated in Fig. S4.

1. **DOA estimation with four-layer S-DNNs**

The performance of the S-DNN for DOA estimation is evaluated with two metrics, i.e., (1) the angular classification accuracy that indicates the confidence value of models, and (2) the angular estimation accuracy evaluated with root mean square error, i.e., RMSE, by using the central angle of angular intervals as the ground truth [s3]. To improve the model performance for testing datasets with complete angular sampling, we further propose the flexible decision boundary to address the misclassification of angles at the angular interval boundary for the single input target. Besides, we generate the multi-target training datasets in addition to the single-input training datasets and further develop the optoelectronic S-DNN architecture for the DOA estimation of multiple input targets, as detailed in the Supplementary Section 8.

We demonstrate that the four-layer S-DNN model can achieve the angular resolution of $\text{1}\text{°}$, which is over four times higher than the diffraction limit resolution at the given angular ranges. The performance of S-DNN for the DOA estimation of single and multiple input targets with the angular resolution of $1^{\circ}$ at the angular range of $\left[ -5^{\circ},5^{\circ} \right]$ and $\left[ 45^{\circ},55^{\circ} \right]$ are shown in Figs. S5 and S6, respectively. For the DOA estimation at the angular range of $\left[ -5^{\circ},5^{\circ} \right]$, the target angle $\theta_{i}$ ($i=0,\ldots,9$) in the $i$-th $1^{\circ}$ angular interval can be formulated as: $\theta_{i}\in\left[ -5^{\circ}+i,-4^{\circ}+i \right]$. The four-layer S-DNN was trained based on the RO3035 PTFE material and the multi-target training dataset, which learns to recognize the target angle $\theta_{i}$ by mapping it to the $i$-th output detection region. The input field distribution, the optical field propagation at a $xz$ plane, and the output plane power distribution evaluated with ASM are demonstrated in Fig. S5a, b, which verifies the correctness of angular recognition and capability of S-DNN for resolving two coherent sources with an angular interval of $\text{1}\text{°}$. Besides the ASM, we also evaluate the performance of S-DNN by utilizing CST Studio Suite for more accurate modeling. The commercial CST Microwave Studio software can perform accurate three-dimensional full-wave EM field simulations based on the time-domain finite integration method to ensure the excellent experimental performance of S-DNN. The material height distribution map of the trained S-DNN is modeled in CST, and PTFE-F4B is utilized as the material with a relative permittivity of $4.03+0.015i$. The CST simulation employs open space boundary conditions, with planewaves of varying incident angles as input and field distribution of S-DNN as output. The simulation frequency range is set to 25~30 GHz. Fig. S5a shows an exemplar inference result of the single target sample with an elevation angle of $-1.5^{\circ}$ at the $3$-th angular interval, and the corresponding CST evaluated result is shown in Fig. S5f (right). Fig. S5b shows an exemplar inference result of the two-target sample with elevation angles of $2.5^{\circ}$ and $3.5^{\circ}$ at the $7$-th and $8$-th angular intervals, respectively. The corresponding output detection regions have the highest intensity measurements. The phase modulation layers of four-layer S-DNN after the training are shown in Fig. S5c, and the corresponding CST model after the full-wave EM field simulation-guided adaptive training is shown in Fig. S5f (left).

The confusion and energy distribution matrices of the four-layer S-DNN model tested on the single-target testing dataset with 10,000 samples, and the two-target testing dataset with 9,000 samples are shown in Fig. S5d, e, respectively. The two targets are at adjacent angular intervals. The results demonstrate the high confidence values, i.e., 99.3% and 99.0%, of the S-DNN model for the DOA estimation of the single target and two-target with $\text{1}\text{°}$ angular resolution, respectively, and the corresponding RMSEs of angular estimation accuracy are $\text{0.23}\text{°}$ and $\text{0.24}\text{°}$, respectively. The average energy percentages of the correct single-target and two-target angular estimation are 34.6% and 29.8%, respectively. For the CST simulation of model performance, considering the computational efficiency and numerical settings in CST software, 100 single-target testing angles were utilized by uniformly sampling at equal intervals within the field-of-view. Each angular interval has 10 testing angles, where the angles around the angular interval boundary with a range of one-tenth of each angular interval were not sampled. The confusion and energy distribution matrices of CST evaluations in Fig. S6g further validate the mode’s effectiveness.

For the DOA estimation at the larger angular range of $\left[ 45^{\circ},55^{\circ} \right]$, we divide the angular range into ten $\text{1}\text{°}$ angular intervals and train the S-DNN model to map the target angles at each angular interval into the corresponding output detection region. The target angle $\theta_{i}$ in the $i$-th angular interval can be formulated as: $\theta_{i}\in\left[ 45^{\circ}+i,46^{\circ}+i \right],i=0,\ldots,9$. The phase modulation layers of the trained four-layer S-DNN are shown in Fig. S6b, and the corresponding CST model with passive intelligent surfaces after the full-wave EM field simulation guided adaptive training is shown in Fig. S6e (left). The ASM and CST evaluation results for recognizing an exemplar input target with an elevation angle of $52.5^{\circ}$ are shown in Figs. S6a and S6e (right), respectively. The confidence values of the S-DNN model evaluated by using the ASM on the single-target testing dataset with 10,000 samples and the two-target testing dataset with 9,000 samples are 97.2% and 98.3%, respectively, and the corresponding RMSEs of angular estimation accuracy are $\text{0.25}\text{°}$ and $\text{0.24}\text{°}$, respectively. The average energy percentages of the correct single-target and two-target angular estimation are 27.5% and 24.9%, respectively. The corresponding confusion and energy distribution matrices of ASM evaluations are shown in Fig. S6c, d. The CST evaluated confusion and energy distribution matrices are shown in Fig. S6f. The results validate the high confidence value of the model to achieve DOA estimation of multiple targets at the angular range of $\left[ 45^{\circ},55^{\circ} \right]$ with $\text{1}\text{°}$ angular resolution. S-DNN achieves the DOA estimation of targets with angular resolution beyond the diffraction limit imposed by the Rayleigh criterion (see Figs. S7 and S8).

1. **Angular resolution upper bound of all-optical S-DNNs**

We evaluate the angular resolution and estimation accuracy upper bound of the S-DNN model for DOA estimation of two targets by increasing the network size and utilizing the CE loss instead of MSE loss during the training. The CE loss facilitates model optimization for higher angular classification accuracy, and the larger network size enables stronger optical field modulation capability of the model for higher performance. The angles of the two targets are set at the adjacent angular intervals during the evaluation, and the confidence value threshold is set to 95% when determining the angular resolution of S-DNN.

The performances of the S-DNN model with respect to the number of modulation elements at each layer under the layer number setting of five layers are shown in Fig. S7a. Obviously, the element number at each layer determines the system aperture size. The results demonstrate that the super-resolved angular resolution (see Fig. S7a, left) and the corresponding angular estimation accuracy evaluated with the RMSE (see Fig. S7a, right) decrease approximately by half with the two times increase of the element number at each layer. The angular resolutions of the S-DNN model for DOA estimation are $0.2^{\circ}$, $0.1^{\circ}$, $0.05^{\circ}$, $0.02^{\circ}$, $0.008^{\circ}$, and $0.004^{\circ}$ for the element numbers of ${16}^{2}$, ${32}^{2}$, ${64}^{2}$, ${128}^{2}$, ${256}^{2}$, and ${512}^{2}$ at each layer, respectively, which are about 40~70 times higher than the Rayleigh limit angular resolutions of $8.74^{\circ}$, $4.37^{\circ}$, $2.18^{\circ}$, $1.09^{\circ}$, $0.55^{\circ}$, and $0.27^{\circ}$, respectively. The angular resolution of $0.004^{\circ}$ correspond to the 14 arc seconds.

We further evaluate the performance of the S-DNN model with respect to the layer number under the modulation element number of $32\times32$ at each layer. Similarly, the angle resolution (see Fig. S7b, left) and the corresponding estimation accuracy (see Fig. S7b, right) evaluated with the RMSE decrease approximately linearly with respect to the layer number before five layers, and the performance of S-DNN suspends to improve when the layer number is larger than five. Multi-layer S-DNN facilitates higher angular resolution and estimation accuracy than the single-layer S-DNN. Since the S-DNN trained with CE loss is more sensitive to systematic errors than MSE loss due to the lower average energy percentage of correction angular estimations, the MSE loss is utilized for the numerical and experimental results in this work. In the future, the in-situ training method can be applied to adapt the model to the systematic errors toward the upper bound performance.

1. **Optical settings of lens systems for comparison**

To compare the system angular response, the lens system is set to have the same optical settings with respect to the S-DNN with a single input target (see Fig. S8). We set the aperture of a lens system to 16$\lambda_{0}$ and the focal length to 5$\lambda_{0}$ with 10 detectors, each with a size of $5\lambda_{0}$/8, arranged sequentially along the elevation direction to measure the elevation angular response. For the incidence plane wave with an elevation angle of 0°, the focusing spot is set to be on the center of the No. 4 detection region. The elevation angle variation of the incident plane wave causes the focusing spot of the lens system to shift continuously along the elevation direction. Since the focusing spot has the Gaussian profile, the DOA estimation of the target can be achieved by finding the center position of the focusing spot. However, the diffraction-limited focusing spot size is ${1.22\lambda_{0}}/{NA}$ for a lens system with a numerical aperture $NA=D/2f$, which places the fundamental limit on angular response sensitivity and results in the diffraction-limited angular resolution of $1.22\lambda_{0}/D$ for two incident plane waves, which is defined as the Rayleigh criterion. We characterize and compare the angular response between S-DNN and lens system at the angular range of $\left[ -5^{\circ},5^{\circ} \right]$. For each incident plane wave, we measure the power value in dB of 10 detection regions at the output plane of the S-DNN and the focal plane of the lens system, respectively, and calculate the power ratio between the two detection regions with the largest and second-largest power values. The power ratio plots of the S-DNN and lens system with respect to the target elevation angles, representing the system angular responses, are provided in Fig. 2d of the main text.

1. **DOA estimation with single-layer S-DNNs**

As shown in Figs. S9 and S10, we train the single-layer S-DNN based on the F4BTME350 PTFE material for determining the target elevation angle with the angular resolution of $\text{10}\text{°}$ at angular range of $[-45^{\circ},55^{\circ}]$. The model learns to classify different target angles in ten angular intervals, i.e., $\theta_{i}\in\left[ -45^{\circ}+10i,-35^{\circ}+10i \right]$, into ten categories, and maps them into the output detection regions. $\theta_{i}$ represents the angles at the $i$-th angular interval with $i=0,\ldots,9$. Fig. S9a shows the numerical inference result of an exemplar input target with an angle of $-28.0^{\circ}$ evaluated with ASM and CST simulations. The optimized phase modulation layer is shown in Fig. S9b with the corresponding CST model shown in Fig. S9d. The input field of the target is mapped to the energy distribution at the output plane, where the No. 1 detection region, corresponding to the angular interval of $\left[ -35^{\circ},-25^{\circ} \right]$, has the max detection energy value for correct categorization. The ASM evaluated confusion matrix and energy distribution matrix, summarized the classification result over 10,000 samples of the testing dataset, are shown in Fig. S9c. The angular classification accuracy of S-DNN, representing the confidence value of all-optical DOA estimation, is 98.7%, and the corresponding RMSE of the model for single-target angular estimation accuracy is $\text{2.6}\text{°}$. More excellent angle estimation accuracy with an RMSE below 0.1° can be achieved based on optoelectronic computing (see Supplementary Section 8). The result also shows the S-DNN model has a high average energy percentage of 79.4% for the correct single-target angular estimation. The CST-evaluated confusion and energy distribution matrices are shown in Fig. S9e, which further validates the effectiveness of the trained S-DNN model.

Besides, we verify the single-layer S-DNN with $\text{10}\text{°}$ angular resolution for simultaneously estimating the elevation angle of two targets at the adjacent angular intervals. Each incident EM wavefront is superimposed by two plane waves from two targets with the elevation angles of $\theta_{i}$ and $\theta_{i+1}$, $i=0,\ldots,8$, from the $i$-th and $(i+1)$-th angular intervals, respectively. Therefore, we generate 9,000 groups of testing samples, each comprising a pair of target angles at the adjacent angular intervals. The output energy distribution of the exemplar testing sample with the target angles of $-41.0^{\circ}$ and $-31.2^{\circ}$ is shown in Fig. S9f, where the corresponding angular intervals, i.e., the 0-th and 1-th, are successfully estimated by finding detection regions with top-two detected energy values. Fig. S9g shows the confusion matrix and energy distribution matrix that summarizes the classification results on 9,000 input samples. The confidence value of DOA estimation for correctly estimating both angles is 98.0%, and the average energy percentage of the correct two-target angular estimation is 43.9%. The corresponding RMSE of the model for two-target angular estimation accuracy is $\text{2.3}\text{°}$.

The experimental results of a single-layer S-DNN for DOA estimation of a single input target are shown in Fig. S10. The fabricated passive S-DNN in Fig. S10a can achieve the broadband DOA estimation. The confidence value of each frequency point is obtained by testing the model on 100 input testing samples uniformly sampling at equal intervals within the field-of-view. Fig. S10b, c show exemplar network outputs and the corresponding energy distribution histogram of detection regions for a single input target with an elevation angle of $-2.5^{\circ}$ at the frequency of 27.5 GHz. The confusion and energy distribution matrices, summarized over 100 testing samples, are shown in Fig. S10d. The model achieves high confidence values above 95% with respect to different testing frequencies between 25 GHz and 30 GHz (see Fig. S10e). The above results validate the effectiveness of S-DNN for DOA estimation at a high confidence value with an angular resolution of $\text{10}\text{°}$ and a field-of-view of $\text{100}\text{°}$.

We further train the single-layer S-DNN for the DOA estimation of a single target with a field-of-view of $\text{150}\text{°}$ and $\text{30}\text{°}$, corresponding to the angular interval of $\text{15}\text{°}$ and $\text{3}\text{°}$, respectively, as shown in Figs. S11 and S12, respectively. For a larger field-of-view, we design a single-layer S-DNN based on the F4BTME350 PTFE material for all-optical DOA estimation with a field-of-view of $[-75^{\circ},75^{\circ}]$, which is divided into ten 15-degree intervals, where the angles $\theta_{i}$ ($i=0,\ldots,9$) in the $i$-th angular interval can be formulated as: $\theta_{i}\in\left[ -75^{\circ}+15i,-60^{\circ}+15i \right]$. The angular spectrum method, CST simulation and experiments in Fig. S11 all demonstrate the effectiveness of S-DNN for DOA estimation with a wide field-of-view of $150^{\circ}$. The optimized phase modulation layer is shown in Fig. S11a. The confusion matrix and energy distribution matrix, summarized the classification result over 10,000 samples of the testing dataset, are shown in Fig. S11b. The angular classification accuracy of S-DNN, representing the confidence value of DOA estimation, is 99.4%, and the corresponding RMSE of the model for single-target angular estimation accuracy is $\text{3.6}\text{°}$. Based on the trained phase profiles for modeling, the CST model of S-DNN is shown in Fig. S11c. We run 100 sets of CST simulations on S-DNN with different plane wave angles at an interval of $\text{1.5}\text{°}$ in the angle range of $[-75^{\circ},75^{\circ}]$. The confusion matrix and energy distribution matrix are shown in Fig. S11d, with an angular classification accuracy of 98%. Based on the 3D model file exported by CST software, the fabricated passive S-DNN is shown in Fig. S11e. We measure the energy values of ten detection regions of S-DNN with 990 different input field angles by controlling the angular rotation stage, carrying the S-DNN to rotate at an interval of $\text{0.15}\text{°}$ within the range of $[-75^{\circ},75^{\circ}]$. S-DNN achieves 97.7% angular classification accuracy, where the confusion matrix and energy distribution matrix are shown in Fig. S11f. Therefore, the S-DNN model trained by the angular spectrum method can show excellent performance in CST simulations and experiments.

Similarly, in Fig. S12, we divide angles in the angular range of $\left[ -15^{\circ},15^{\circ} \right]$ into ten 3-degree intervals, where the angles $\theta_{i}$ ($i=0,\ldots,9$) in the $i$-th angular interval can be formulated as: $\theta_{i}\in\left[ -15^{\circ}+3i,-12^{\circ}+3i \right]$. After the network training based on the F4BTMS350 PTFE material, Fig. S12a shows an input field and the correct inference results, verified with the ASM and CST numerical model, for an exemplar input target with an angle of $-13.5^{\circ}$. The No. 0 detection region with a white square label, corresponding to the correct angle interval of $\left[ -15^{\circ},-12^{\circ} \right]$, has the highest detection energy. The phase modulation layer of the trained single-layer S-DNN is shown in Fig. S12b. The corresponding CST model and the fabricated single-layer S-DNN are shown in Figs. S12d, f, respectively. The ASM evaluated confusion matrix and energy distribution matrix of the trained model, tested over 10,000 samples, are shown in Fig. S12c. The confidence value of the S-DNN model for single-target DOA estimation with $\text{3}\text{°}$ angular interval is 99.2%, and the average energy percentage of the correct single-target angular estimation is 45.7%. The corresponding RMSE of the model for single-target angular estimation accuracy is $\text{0.73}\text{°}$. The CST evaluated and experimental confusion and energy distribution matrices are shown in Fig. S12e, g, respectively, which further validates the effectiveness of the trained S-DNN model. Besides, the single-layer S-DNN for $\text{4°}$ angular resolution can achieve the super-resolution DOA estimation of two target sources from arbitrary angular intervals with an angular classification accuracy of 95.1% with an average energy percentage of 31.4% (see Fig. S13).

1. **DOA estimation with three-layer S-DNNs**

To improve the confidence value of DOA estimation for multiple input targets, we construct the three-layer S-DNN to achieve the $\text{3}\text{°}$ angular resolution beyond the diffraction limit within the field-of-view of $\left[ -15^{\circ},15^{\circ} \right]$. The DOA results of two-target at adjacent angular intervals and separated by one angular interval are shown in Fig. S14. After the network training of three-layer S-DNN based on the F4BTME350 PTFE material, the diffractive modulation layers are shown in Fig. S14b, and three exemplar inference results are shown in Fig. S14a, c, e. The confusion matrices and energy distribution matrices of the S-DNN model tested on the two types of testing datasets that include two targets at adjacent angular intervals and separated by one angular interval are shown in Fig. S14d, f, respectively. The confidence values of super-resolution DOA estimation on the two types of the two-target testing datasets are 95.7% and 99.5%, respectively, where the corresponding RMSEs of angular estimation accuracy are $\text{0.81}\text{°}$ and $\text{0.77}\text{°}$, respectively. The average energy percentages of the correct two-target angular estimation are 40.6% and 34.9%, respectively.

We also evaluate the all-optical DOA estimation performance of S-DNN for two-target with arbitrary angles of $\theta_{i}$ and $\theta_{j}$ from the $i$-th and $j$-th angular intervals, respectively, where $i=0,\ldots,8$ and $j=1,\ldots,9$ with $j>i$. We generate 9,000 groups of two-target testing samples, which include a total of 45 permutations of different angular intervals, each containing 200 samples with a random angle combination of two targets. The confusion and energy distribution matrices of the model evaluated on the two-target testing datasets are shown in Fig. 3g of the main text and Fig. S15, respectively. The confidence value of the S-DNN model for super-resolution DOA estimation with arbitrary angles of two targets is 94.9%, where the corresponding RMSE of angular estimation accuracy is $\text{0.88}\text{°}$. The average energy percentage of the correct angular estimation is 33.7%. Besides, the experimental results evaluated in a microwave anechoic chamber further demonstrate excellent broadband DOA estimation performance of the three-layer S-DNN between the frequency range of 25 GHz and 30 GHz (see the main text). For an optical system with a wavelength of $\lambda_{0}$ and an aperture size of $D$, the Rayleigh criterion defined diffraction-limited angular resolution can be formulated as: $1.22\lambda_{0}/D$, which is $4.37^{\circ}$ with the setting of $D=16\lambda_{0}$. The S-DNN is able to generate the super-oscillated angular response at the local angular range of $\left[ -15^{\circ},15^{\circ} \right]$ and achieves the super-resolved angular resolution of $\text{3}\text{°}$.

1. **All-optical S-DNNs for DOA estimation with flexible decision boundary**

With the fixed decision boundary by finding the max intensity or power measurement among ten detection regions, the angles at interval boundaries are easily misclassified into their adjacent intervals, resulting in a decrease in the classification accuracy. To address this issue, we propose the flexible decision boundary strategy by comparing the ratio of top-two power measurements to the pre-calibrated decision coefficients, which can effectively improve the classification accuracy of S-DNN by correctly classifying angles at the interval boundary. For an incident plane wave from a target with an unknown angle, we identify two detection regions with the highest and second highest detected power, i.e., $P_{i}$ and $P_{j}$ ($i<j$), corresponding to the $i$-th and $j$-th angular intervals, respectively. If two detection regions are not adjacent, the angular interval label of the target is determined by using the detection region with max power. If two detection regions are adjacent with $j=i+1$, indicating that the target angle is more likely to be at the angular interval boundary, we calculate the power ratio of two detection regions, i.e., $log(P_{i}/P_{i+1})=log(P_{i})-\log{(P}_{i+1})$, and compare it to the pre-calibrated decision coefficients $\xi_{i,i+1}$. $\xi_{i,i+1}$ is obtained by measuring and calculating the power ratio of adjacent detection regions with the input target at the intersected angle of adjacent angular intervals. The target is classified into the *i*-th angular interval if the power ratio is larger than $\xi_{i,i+1}$; otherwise, the target is classified into the (*i*+1)-th angular interval.

Fig. S16 illustrates the use of flexible decision boundary to improve the testing accuracy, i.e., confidential value, of S-DNN model for the DOA estimation of target elevation angles with a field-of-view of $\left[ -5^{\circ},5^{\circ} \right]$ and an angular interval range of $1^{\circ}$. The S-DNN is configured with four layers and the field-of-view is divided into ten angular intervals, where the angles at the $i$-th angular interval can be formulated as: $\theta_{i}\in\left[ -5^{\circ}+i,-4^{\circ}+i \right]$ with $i=0,\ldots,9$. We calculate the power ratio between two successive detection regions, i.e., $\log\left( P_{i}/P_{i+1} \right)$, with $P_{i}$ and $P_{i+1}$ being the top-two detected power values, for each target angle. The plots on the numerical testing dataset with 10,000 samples are shown in Fig. S16a, b, and the experimental testing dataset with 1,000 samples are shown in Fig. S16c, d. The experimental testing samples are uniformly generated by rotating the passive S-DNN, which is achieved by utilizing the angular rotation stage in the microwave anechoic chamber with an angular rotation resolution of $0.01^{\circ}$. With the fixed decision boundary of $\xi_{i,i+1}=0$ for comparisons, we can find that there are large numbers of misclassification angles around angular interval boundaries indicated with green dots, resulting in the low testing accuracies of 93.4% and 91.6% for the numerical and experimental results, respectively. In contrast, with the pre-calibrated flexible decision coefficients $\xi_{i,i+1}$ for comparison, the misclassification angles are dramatically eliminated, and the testing accuracies are substantially enhanced to 99.6% and 95.8% for the numerical results and experimental results under the detection noise, respectively. Both numerical and experimental results demonstrate that the flexible decision boundary strategy can effectively improve the confidence value of S-DNN for DOA estimation.

1. **DOA estimation with optoelectronic S-DNNs**

An optoelectronic S-DNN architecture is proposed to further improve the angle estimation accuracy of all-optical S-DNN, which can greatly enhance the angle estimation accuracy while ensuring the wide field-of-view without additional space-time multiplexing mechanism. The optoelectronic S-DNN architecture requires the detected energy values of S-DNN for plane waves at different angles to construct the energy-angle characteristic curve of S-DNN as prior information, and uses the least square method (LSM) to find the prior angle that best matches the energy response of the unknown target. The optoelectronic S-DNN requires only a small amount of electronic computing to significantly improve the DOA estimation performance of S-DNN, in terms of RMSE and the confidence value of angular resolution.

To experimentally verify the effectiveness of optoelectronic S-DNN in the single-target estimation task, we fabricate the three-layer passive S-DNN with a field-of-view of $\left[ -15^{\circ},15^{\circ} \right]$ using the trained phase pattern (see Fig. S14b). We establish an experimental system in the microwave anechoic chamber to collect prior information to construct the energy-angle characteristic curve of S-DNN, which is controlled by the angle turntable to achieve rotation (see Fig. S4). We uniformly gather the energy values of ten detection regions as prior information when plane waves incident at 121 different angles at an interval of 0.25° within the range of $\left[ -15^{\circ},15^{\circ} \right]$. Then the cubic interpolation method is adopted to fit the energy-angle characteristic curve of S-DNN in the whole angle range by interpolating the energy values of 121 discrete angles, as shown in Fig. S17a. Obviously, the energy-angle characteristic curve describes the energy response of S-DNN to target sources at different angles, that is, the energy value of the detection region corresponding to the angle interval where the target is located is the highest. The ten energy curves in Fig. S17a constitute a ten-dimensional energy space, and the ten energy values of different incident angles can be regarded as the coordinates of the corresponding angle in the ten-dimensional energy space. For an unknown plane wave as the input of S-DNN, the energy values of the ten detection regions also correspond to an arbitrary point in the ten-dimensional space. Based on the LSM, we can calculate the prior point with the closest Euclidean distance to the point corresponding to the input plane wave, and then find the corresponding angle on the energy-angle characteristic curve as the DOA estimation result. Fig. S17b shows the experimental comparison of the angle estimation results of optoelectronic S-DNN and all-optical S-DNN. Since the all-optical S-DNN can only classify the incident plane wave into the corresponding angle interval and utilize the intermediate value of this angular interval as the angle estimation result, the curve presents a staircase shape. On the other hand, optoelectronic S-DNN uses prior information to obtain the estimated angle, which is almost equal to the incident angle. Experimental results confirmed that the optoelectronic S-DNN achieves an angle estimation accuracy with an RMSE of $0.018^{\circ}$ for 1,000 random incident angles within $\left[ -15^{\circ},15^{\circ} \right]$, while the RMSE of the all-optical S-DNN is $0.85^{\circ}$. Additionally, the optoelectronic S-DNN is applicable to larger field-of-view to significantly improve the RMSE metric of angular estimation accuracy.

Numerical simulation proves that a three-layer S-DNN using an optoelectronic estimation approach can effectively improve the confidence value of angular resolution in two-target DOA estimation tasks. We collect the energy values of the detection regions as prior information, when the input of S-DNN is the superposition of two plane waves from two targets with the elevation angles of $\theta_{i}$ and $\theta_{j}$, $i=0,\ldots,8$, $j=1,\ldots,9$ from the $i$-th and $j$-th angular intervals, respectively. Therefore, we generate 9,000 groups of two-target samples as prior information, and there are a total of 45 permutations of different angular intervals, each containing 200 random angles. After obtaining the energy-angle characteristic curve of S-DNN, we generate 9,000 sets of testing samples, including two targets distributed at arbitrary angular intervals, to verify the effectiveness of optoelectronic S-DNN. The confusion matrices and energy distribution matrices are shown in Fig. S17c. The confidence value of angular resolution on the testing datasets, including all permutations of two targets, is 99.5%, where the corresponding RMSE of angular estimation accuracy are $0.033^{\circ}$.

1. **Integrated sensing and communication with reconfigurable S-DNNs**

We proposed a system architecture of integrated sensing, computing, and communication based on the reconfigurable S-DNN consisting of a single reflective LC RIS, as shown in Fig. 4 of main text. Reconfigurable S-DNN utilizes the programmability and high phase modulation accuracy of LC RIS, and adopts time-division multiplexing mechanism to realize DOA estimation of mobile users and establish a real-time communication link between users and base station.

For the azimuth DOA estimation task, we design the reconfigurable S-DNN with a field-of-view of $\left[ -15^{\circ},15^{\circ} \right]$, which is divided into ten angular intervals, corresponding to ten detection regions arranged sequentially along the azimuth direction on the output plane. Since the reconfigurable S-DNN works in reflection mode, we set the detection regions obliquely in front of the S-DNN, $15\lambda$ away from the S-DNN, to avoid the occlusion between the detector array and the incident wave (see Fig. S2b). Since the reconfigurable S-DNN is sensitive to elevation angle variations caused by antenna placement errors, we add random elevation angles in the range of $\left[ -8^{\circ},8^{\circ} \right]$ to the training dataset to improve the robustness of the reconfigurable S-DNN to the elevation angle variations, so as to achieve the accuracy of azimuth angle estimation (see Methods of the main text). The trained DOA estimation phase is shown in Fig. 4e of main text. The confusion matrix and energy distribution matrix of the trained S-DNN, tested over 10,000 samples, are shown in Fig. S18a. The confidence value of the reconfigurable S-DNN model for single-target DOA estimation is 97.7%, and the average energy percentage of the correct single-target angular estimation is 51.3%. In the experiment, we utilize the optoelectronic method to improve the angular estimation accuracy of the reconfigurable S-DNN (see Supplementary Section 8). We uniformly collect the energy values of detection regions as prior information when plane waves incident at 60 different angles within the range of $\left[ -15^{\circ},15^{\circ} \right]$. Experimental results shown in Fig. S18b demonstrate that the reconfigurable S-DNN based on the optoelectronic method achieves an angular estimation accuracy with an RMSE of $0.19^{\circ}$ for 60 random incident angles within $\left[ -15^{\circ},15^{\circ} \right]$.

With the prior angle information $\theta_{1}$ of the base station, the reconfigurable S-DNN utilizes the estimated angle $\theta_{2}$ of the user to optimize the beamforming phase:

$$P\left( x, y,\lambda\right)=\left\{ \frac{2\pi}{\lambda}(x(\sin\theta_{1}\cos\varphi-\sin\theta_{2}\cos\varphi)+y(\sin\theta_{1}\sin\varphi-\sin\theta_{2}\sin\varphi)) \right\}*\frac{180}{\pi} \text{(5)}$$

where $(x,y)$ denotes the coordinate of the modulation elements of RIS; $\varphi$ refers to the elevation angle of the base station and user, which is set to be $0^{\circ}$ in the experiment. Fig. S18c shows the beamforming phase of an exemplar testing sample with the $\theta_{1}$ and $\theta_{2}$ setting to be $-7^{\circ}$ and $12^{\circ}$, respectively. By loading the optimized beamforming phase, the reconfigurable S-DNN can reflect the transmitted EM waves of the base station to the user to establish a real-time communication link.

1. **All-optical source number estimation diffractive neural networks**

Source number estimation denotes the measurement of the target source number in free space, which is an important technology in wireless signal processing and can provide prior information for subsequent algorithms, such as DOA estimation, beamforming, and blind source separation [s4]. Based on the ability to perceive the direction of EM waves, S-DNN can realize all-optical source number estimation for multiple coherent sources by re-optimizing the network architecture, as shown in Fig. S19a.

We design a five-layer S-DNN with an elevation field-of-view of $\left[ -45^{\circ},55^{\circ} \right]$ for estimating the number of target sources at different angular intervals, where the input phase distribution of S-DNN is the superposition of multiple plane waves. After multiple layers of phase modulation and free-space diffractive propagation, S-DNN accumulates the superimposed input field into the detection region corresponding to the number of target sources. The number of target sources corresponding to the No. $i$ detection region is $i=1,\ldots,10$, which means that S-DNN can simultaneously estimate up to ten target sources. The optimized five phase modulation layers are shown in Fig. S19a. For an exemplar consisting of five target sources at different angular intervals, the output plane and energy distribution histogram demonstrate the No.5 detection region has the highest energy value, i.e., the number of target sources is five. The confusion matrix and energy distribution matrix summarize the classification results for the 5,000 testing samples, including 10 multi-target scenes, the number of targets ranges from 1 to 10, and each scene contains 500 combinations of random angles. From Fig. S19b, S-DNN can achieve all-optical source number estimation for coherent sources with a 98.8% confidence value, and an average energy percentage of up to 33%.

Therefore, S-DNN performs wireless signal processing tasks such as source number estimation, DOA estimation, and beamforming at the speed of light by switching the loading phase profiles rapidly. S-DNN is expected to replace commercial software-defined radio processors, or offload part of the computational burden from the base station to the edge devices.

1. **Comparison of S-DNN with MUSIC DOA estimation method**

To demonstrate the advantages of S-DNN, we compare the system architecture and the angular resolution of DOA estimation between S-DNN and conventional architecture based on an electronic computing processor for running a multiple signal classification (MUSIC) algorithm [s5].

The conventional DOA estimation methods are based on the architecture of separating signal acquisition and processing, where the signal acquisition utilizes the phased array system with $N$ modules, and the captured multi-channel signals are processed with a digital signal processor (DSP), as illustrated in Fig. S1a. Each module of signal acquisition comprises the electronic elements of the antenna, phase shifter, RF mixer, and analog-to-digital converter. The radio-frequency (RF) signals are first coupled into the antenna array and modulated with phase shifters, then down-converted with RF mixers and analog-to-digital converted with ADC to obtain the baseband signals. The utilization of costly hardware systems, massive amounts of sampled data, and complex signal processing algorithms result in high sensing latency, high cost, and high-power consumption of DOA estimation. The proposed S-DNN is based on the all-optical diffractive neural networks composed of multi-layer reconfigurable or passive intelligent surfaces and detection arrays, enabling high-dimensional modulations of the incident optical fields. Compared with electronic DOA estimation methods, S-DNN achieves integrated in-memory sensing and computing with substantially reduced power consumption and latency.

We compare the angular resolution between S-DNN and the MUSIC algorithm to demonstrate the superior performance of S-DNN for DOA estimation, as shown in Fig. 4e of the main text. MUSIC algorithm requires measuring multiple snapshots to utilize the temporal information of baseband signals for estimating the angles of incoherent target sources. In contrast, the S-DNN only requires a single snapshot measurement that utilizes the spatial distribution of the incident EM field for estimating the angles of both coherent and incoherent target sources. The fundamental principle of the MUSIC algorithm utilizes the orthogonality of the noise subspace and the signal subspace as well as the identity of the steering vector and the signal subspace, to estimate the angles of incoherent targets. The spatial spectrum of the MUSIC algorithm is formulated as follows:

$P_{MUSIC}\left( \theta\right)=\frac{1}{\left| a^{H}(\theta)GG^{H}a(\theta) \right|}$ (6)

where $a(\theta)$ and $G$ denote the steering vector and noise subspace, respectively; $\left( \cdot\right)^{H}$ denotes the conjugate transpose operator. With the angle scanning, the target angle can be determined by finding the peak value of the calculated spatial spectrum. During the evaluations, the system parameters of the electronic DOA estimation system running the MUSIC algorithm are set to be the same with respect to the S-DNN, where the antenna array dimension is set to be 32 with half-wavelength unit size, operating at a frequency of 27.5 GHz. Both S-DNN and MUSIC are applied to estimate the angles of two targets located at the central field-of-view, where the conclusions also hold for the DOA estimation at larger angles.

Under the input SNR of 10 dB, the estimated angular resolutions of the MUSIC algorithm are $\text{5}\text{°}$ and $\text{2}\text{°}$ with 2 and 100 snapshots, respectively (see Fig. 4e of the main text). The MUSIC algorithm fails for the angular estimation with a single snapshot measurement. For comparison, the S-DNN only requires a single snapshot measurement and achieves the angular resolution of $\text{1}\text{°}$ and $\text{0.1}\text{°}$ with the MSE loss and cross-entropy loss, respectively. For the 100 snapshots, the angular resolutions of the MUSIC algorithm are decreased and increased to 0.5° and 4° with the input SNR of 30 dB and 0 dB, respectively. For comparison, the S-DNN is more robust to the input noise and achieves an angular resolution of 0.2° even when the input SNR is reduced to 0 dB. The comparison results demonstrate the performance of the MUSIC algorithm substantially decreases with the decrease of snapshots and SNRs, while the S-DNN can achieve higher angular resolution without requiring the temporal sampling of baseband signals and is more robust to input noise.

1. **Computing performance of passive S-DNNs**

We calculate the computing speed and energy efficiency of the passive S-DNN architectures based on the sampling time and power consumption, respectively. The optical computational operations consist of optical field modulation, diffractive weighted interconnection, and complex nonlinear activation. During the calculation, the number of complex operations in the S-DNN is converted to the number of real operations, where each complex multiplication is equivalent to 4 real multiplications and 2 real summations, and each complex summation is equivalent to 2 real summations. With the number of modulation elements per PIS layer is $K$, the number of optical real operations contained in both optical field modulation and complex nonlinear activation is $6K$. Diffractive weighted interconnection based on the angular spectrum method (ASM) comprises the FFT, dot product, and IFFT, where there are in total $2K{log}_{2} \sqrt{K}+K$ complex multiplications and $4K{log}_{2} \sqrt{K}$ complex summations, corresponding to the real operations of $20K{log}_{2} \sqrt{K}+6K$. Considering that the S-DNN contains four layers of PIS, the total number of optical real operations is $80K{log}_{2} \sqrt{K}+54K$. The computing speed that measures the total number of operations per second of the S-DNN can be formulated as:

$$v=\frac{R_{o}}{t_{s}+t_{p}}\approx\frac{R_{o}}{t_{s}} \text{(7)}$$

where $R_{o}$, $t_{s}$, and $t_{p}$ denote the total number of optical real operations, the sampling time of the detector, and the propagation time of the optical field from the input plane to the output plane, respectively. In Eq. (7), we ignore $t_{p}$ during the calculation as it is less than 1 *ns* for the constructed four-layer S-DNN in this work.

Computing energy efficiency measures the total number of operations per watt-second for the S-DNN and consists of two metrics, direct energy efficiency and system energy efficiency. Direct energy efficiency is calculated based on the energy consumption directly used for optical computational operations of all-optical S-DNN, indicating a potential upper limit of processor’s energy efficiency. The system energy efficiency is calculated based on the energy consumption of the experimental prototype system and represents the energy efficiency of the all-optical S-DNN with electronic peripherals in this work. The direct energy efficiency only considers the optical field energy consumption during the propagation of the incident optical field through the S-DNN to the output plane, where the detection regions typically receive 1% of the incident optical field energy. Therefore, the incident optical field energy can be approximated as the energy consumption of the S-DNN. The energy of detector regions can be measured by a commercial power probe with a minimum detected power of -50 dBm, i.e., $1\times{10}^{-5}$ *mW*, corresponding to the minimum incident optical field power $P_{o}$ of $1\times{10}^{-3}$ *mW*. The direct energy efficiency can then be formulated as:

$$\rho_{direct}=\frac{R_{o}}{E_{o}} \text{(8)}$$

where $E_{o}=P_{o}t_{s}$ denotes the optical field energy consumption of S-DNN during measurement. We calculate the system energy efficiency by incorporating the energy consumption of all electronic peripherals in the experimental system as follows:

$$\rho_{system}=\frac{R_{o}}{E_{e}}=\frac{R_{o}}{(P_{VNA}+P_{Desktop})t_{s}} \text{(9)}$$

We set the $t_{s}$ to be the VNA sampling time, i.e., $t_{s}=67 ns$, to evaluate the upper bound performance. During the experiment, the modulation elements number $K=32\times32$, and the power consumption of VNA and Desktop are: $P_{VNA}=90 W, P_{Desktop}=100 W$. Therefore, the calculated computing speed, direct energy efficiency, and system energy efficiency of passive S-DNN are 6.94 TOPs s^-1^ (tera-operations per second), 6.94 POPs J^-1^ (exa-operations per watt-second), and 36.5 GOPs J^-1^, respectively. The network size of S-DNN can be further increased to facilitate higher angular resolution (see Fig. S7a). The five-layer S-DNN, each layer with $K=512\times512$ modulation elements, can reach the angular resolution of $0.004^{\circ}$ with CE loss. The corresponding computing speed and system energy efficiency are 3.78 POPs s^-1^ and 19.9 TOPs J^-1^, respectively. Note that the cutting-edge Nvidia Tesla V100 GPU has a computing speed of ~30.0 TOPs s^-1^ and a system energy efficiency of ~0.1 TOPs J^-1^ [s6]. The network size of passive S-DNN can be easily scaled up for DOA estimation with higher angular resolution and facilitating advanced computing speed and energy efficiency over cutting-edge GPUs. Besides, the estimation latency of ~$67 ns$ with VNA substantial advantages over existing electronic radio direction-finding devices.


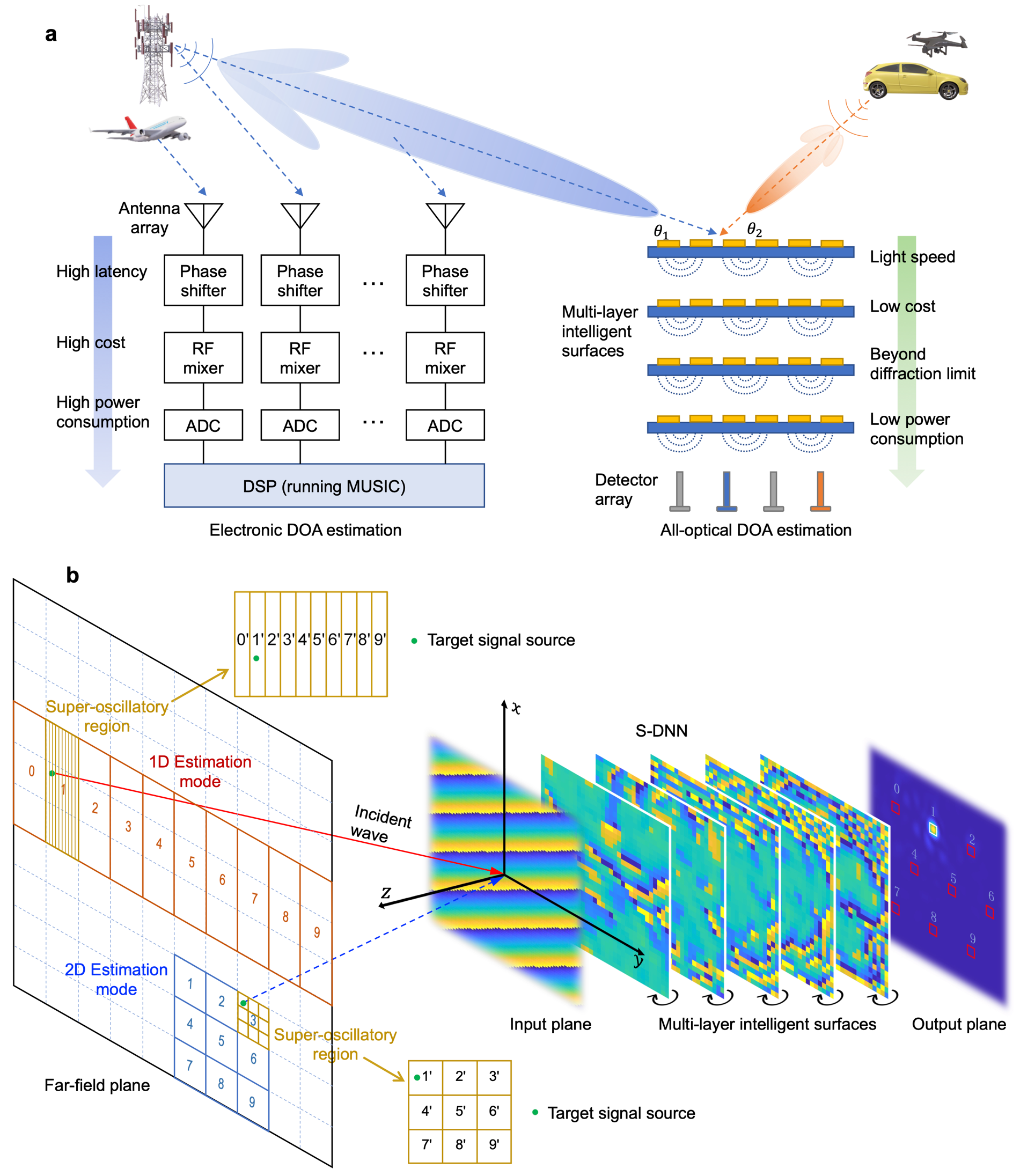


**Figure S1. The principle of S-DNN for DOA estimation under 1D or 2D mode and its comparisons with electronic DOA estimation. a**, System diagram comparisons demonstrate the substantial advantages of all-optical S-DNN architecture over conventional electronic methods for target DOA estimation. **b**, S-DNN is constructed by cascading multiple intelligent surfaces, which maps input angular intervals into corresponding output detection regions. S-DNN can generate super-oscillatory angular responses in local angular regions. Different S-DNN models can be trained to perform the DOA estimation of targets with different field-of-views and angular resolutions under 1D or 2D estimation mode.


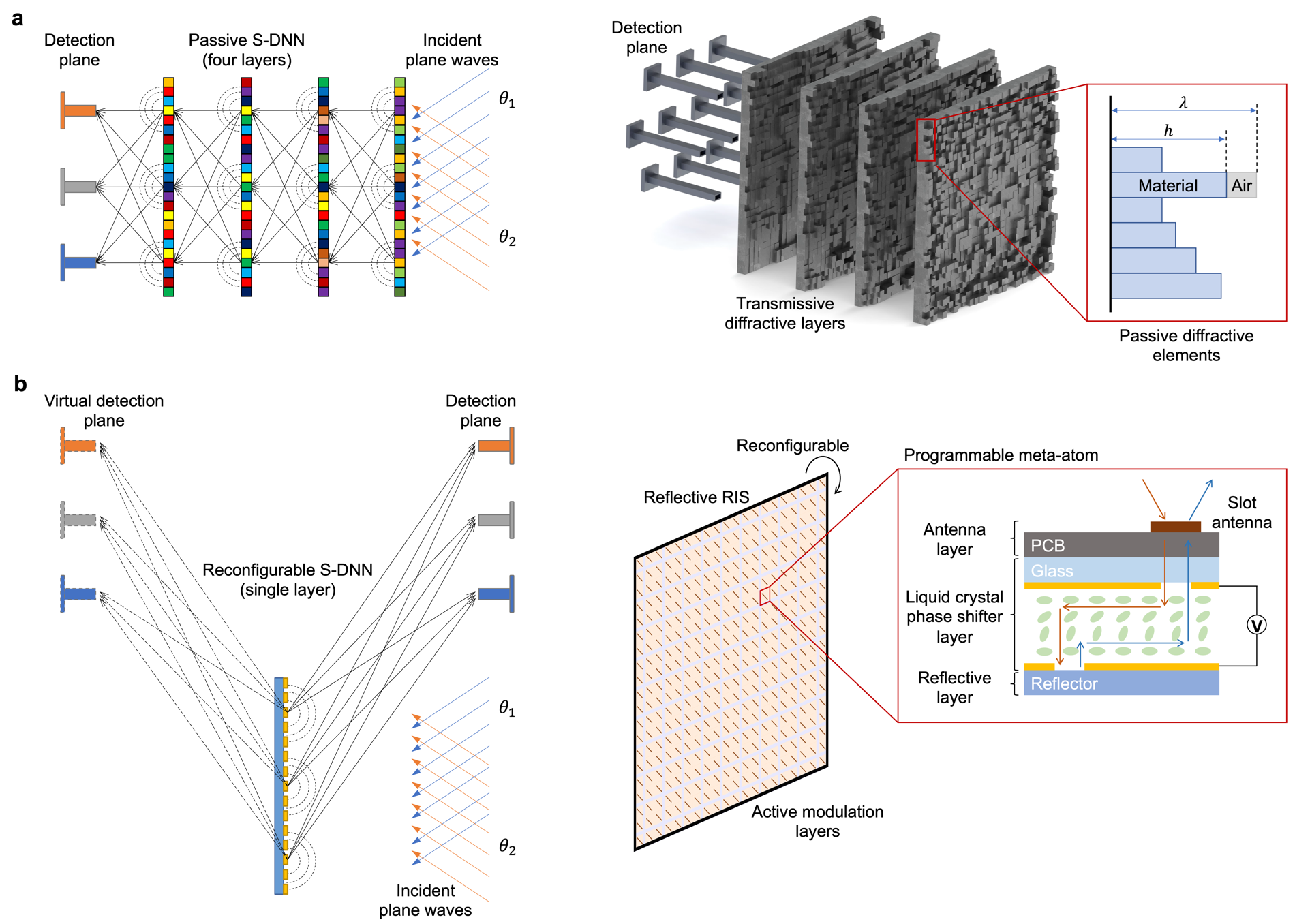


**Figure S2. Constructing S-DNN with passive or reconfigurable diffractive layers. a**, The passive S-DNN comprises the cascading of multiple passive transmission layers. The diffractive elements at each layer modulate the phase of the incident plane wave and generate a spherical secondary wave that interconnects to the diffractive elements of the next layer through optical diffractions. The transmission coefficients of the diffractive elements can be trained by deep learning, enabling S-DNN to perform the DOA estimation task. The diffractive layer of passive S-DNN is made of PTFE dielectric material with high-precision CNC machining. The passive diffractive elements are accurately modeled considering the multi-beam interference effect. **b**, The reconfigurable S-DNN is composed of reflective RIS, which can dynamically update the phase modulation coefficient to switch models for DOA estimation at different angle ranges. The liquid crystal RIS adjusts the deflection direction of the liquid crystal by controlling the voltage, which changes the dielectric constant of the liquid crystal layer and achieves phase modulation.


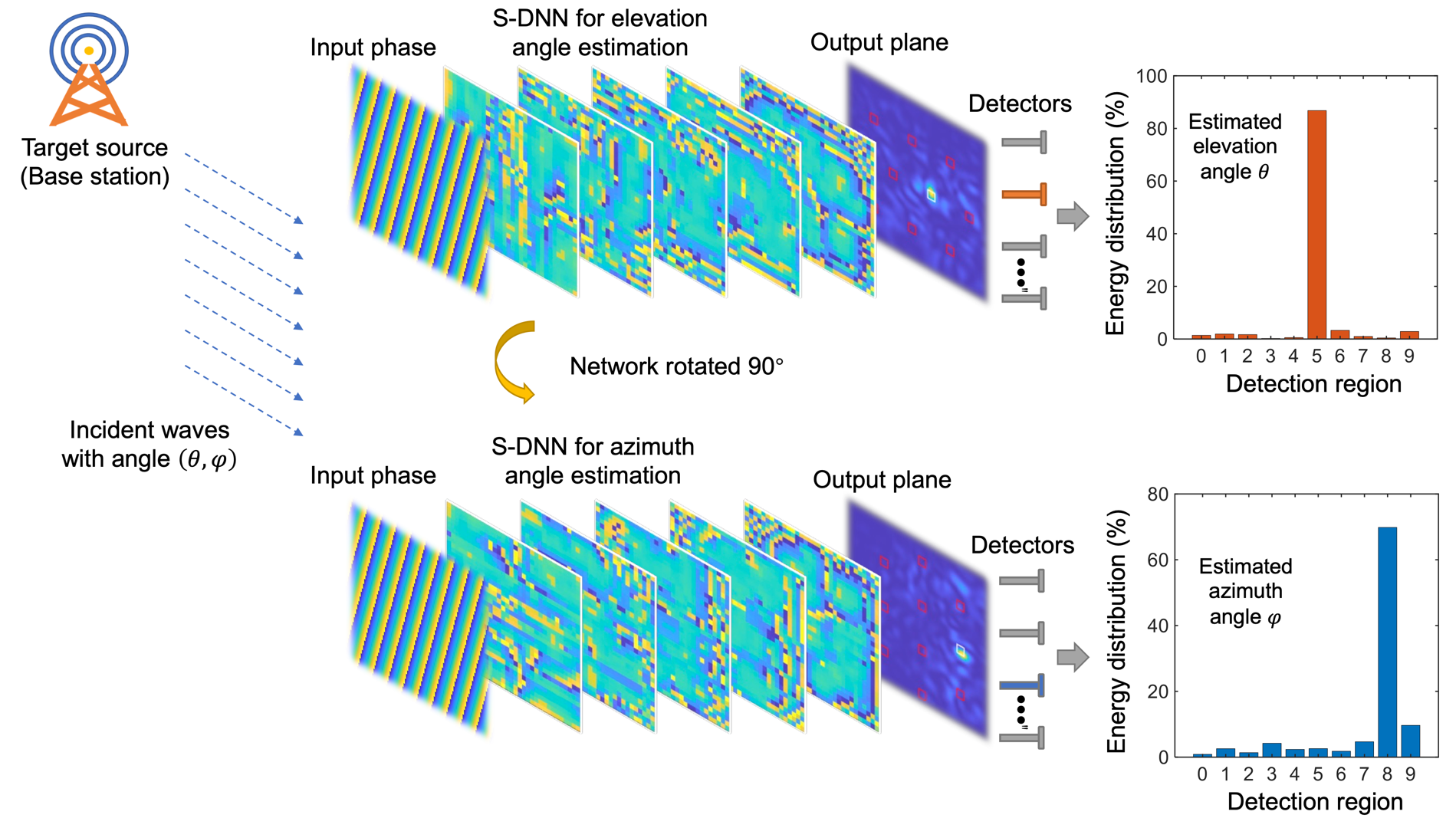


**Figure S3. S-DNN with 1D estimation mode for separately estimating the elevation and azimuth angles.** The elevation and azimuth angles are orthogonal directions in 3D space. Therefore, the S-DNN trained for elevation angle estimation can be utilized for azimuth angle estimation by rotating the network at 90 degrees. This also means that the S-DNN trained for elevation angle estimation can be experimentally verified with the azimuth-rotation system after rotating the network at 90 degrees. The exemplar network is trained for the DOA estimation at the angular range of $[-45^{\circ},55^{\circ}]$ with an angular resolution of $10^{\circ}$. Thus, for the exemplar incident plane wave from a target source with an elevation angle of $10^{\circ}$ and azimuth angle of $40^{\circ}$, the No. 5 detection region at the output plane, corresponding to an elevation angular interval of $[5^{\circ},15^{\circ}]$, obtains the max intensity measurement. The azimuth angle estimation can further be obtained by rotating the trained S-DNN at 90°, where the No. 8 detection region, corresponding to the azimuth angular interval of $[35^{\circ},45^{\circ}]$, obtains the maximum intensity measurement.


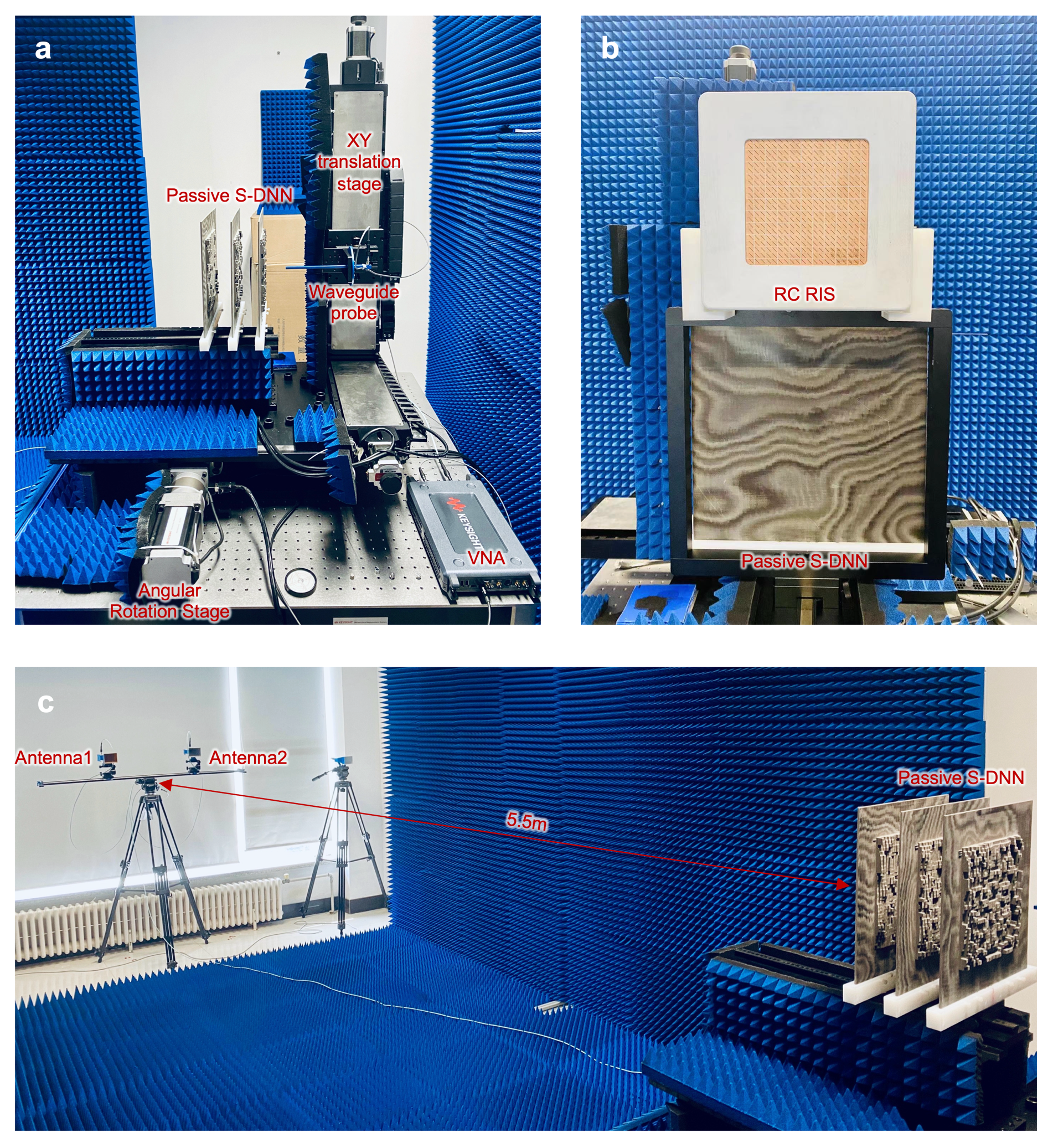


**Figure S4. Experimental system.** The experimental system for characterizing and measuring the output field distributions of S-DNN to achieve the super-resolution DOA estimation with the application for low-latency RIS-based communication.


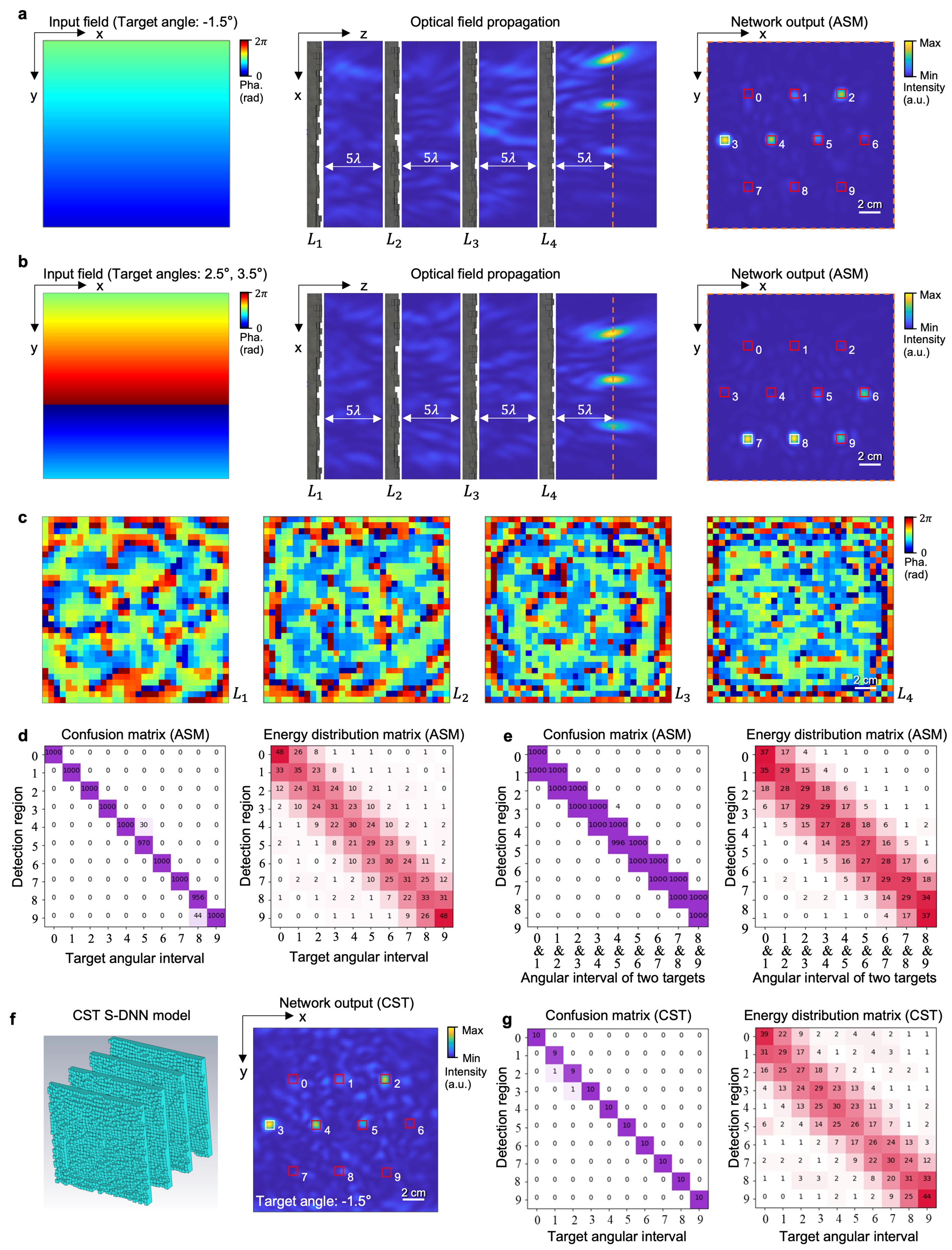


**Figure S5. Numerical results of four-layer S-DNN for DOA estimation with** $\text{1}\boldsymbol{^{\circ}}$ **angular resolution at the angular range of** $\boldsymbol{[-5}\boldsymbol{^{\circ}}\boldsymbol{,5}\boldsymbol{^{\circ}}\boldsymbol{]}$**. a**, **b**, The input field distributions, optical field propagations, and output power distributions of two exemplar inference results with single and two input targets. **c**, Phase distribution of four diffractive modulation layers after the training. **d**, **e**, The confusion matrices and energy distribution matrices evaluated on the single-target and two-target testing datasets. **f**, The CST model of four-layer S-DNN with passive layers and an exemplar inference result of the target elevation angle of $-1.5^{\circ}$. **g**, The confusion and energy distribution matrices evaluated by using CST simulation on the single-target testing datasets.


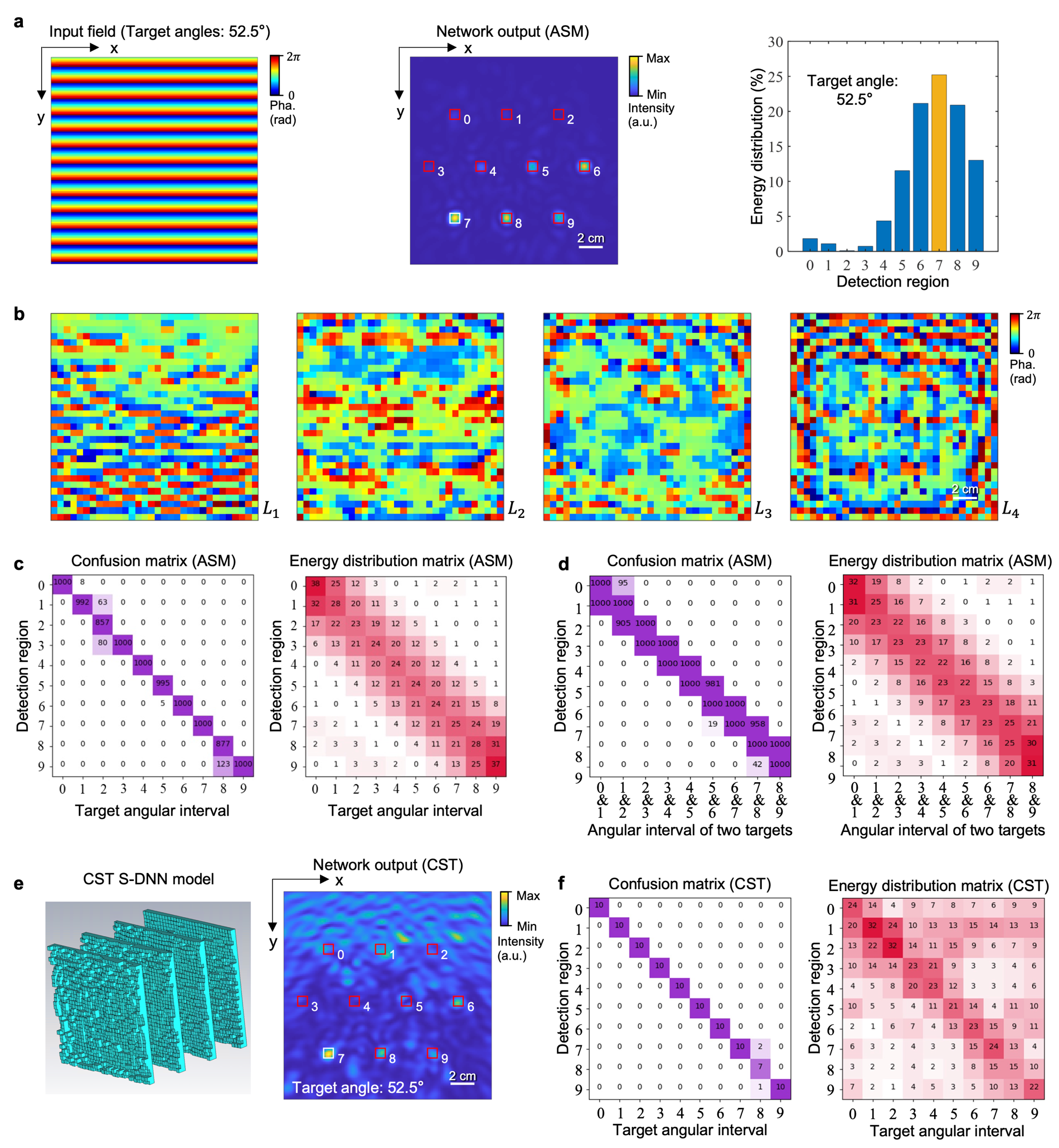


**Figure S6. Numerical results of four-layer S-DNN for DOA estimation with** $\text{1}\boldsymbol{^{\circ}}$ **angular resolution at the angular range of** $\boldsymbol{[45}\boldsymbol{^{\circ}}\boldsymbol{,55}\boldsymbol{^{\circ}}\boldsymbol{]}$**. a**, The exemplar inference result, evaluated by using ASM, of a target with an elevation angle of $52.5^{\circ}$. **b**, Phase patterns of four diffractive modulation layers after the training. **c**, **d**, The confusion matrices and energy distribution matrices of the four-layer S-DNN model, evaluated by using ASM, on the single-target and two-target testing datasets at the angular range of $[45^{\circ},55^{\circ}]$. **e**, The CST model of four-layer S-DNN with passive layers and an exemplar inference result, evaluated by using CST simulation, of the same target with an elevation angle of $52.5^{\circ}$. **f**, The confusion matrix and energy distribution matrix of the four-layer S-DNN model, evaluated by using CST simulation, on the single-target testing datasets at the angular range of $[45^{\circ},55^{\circ}]$.


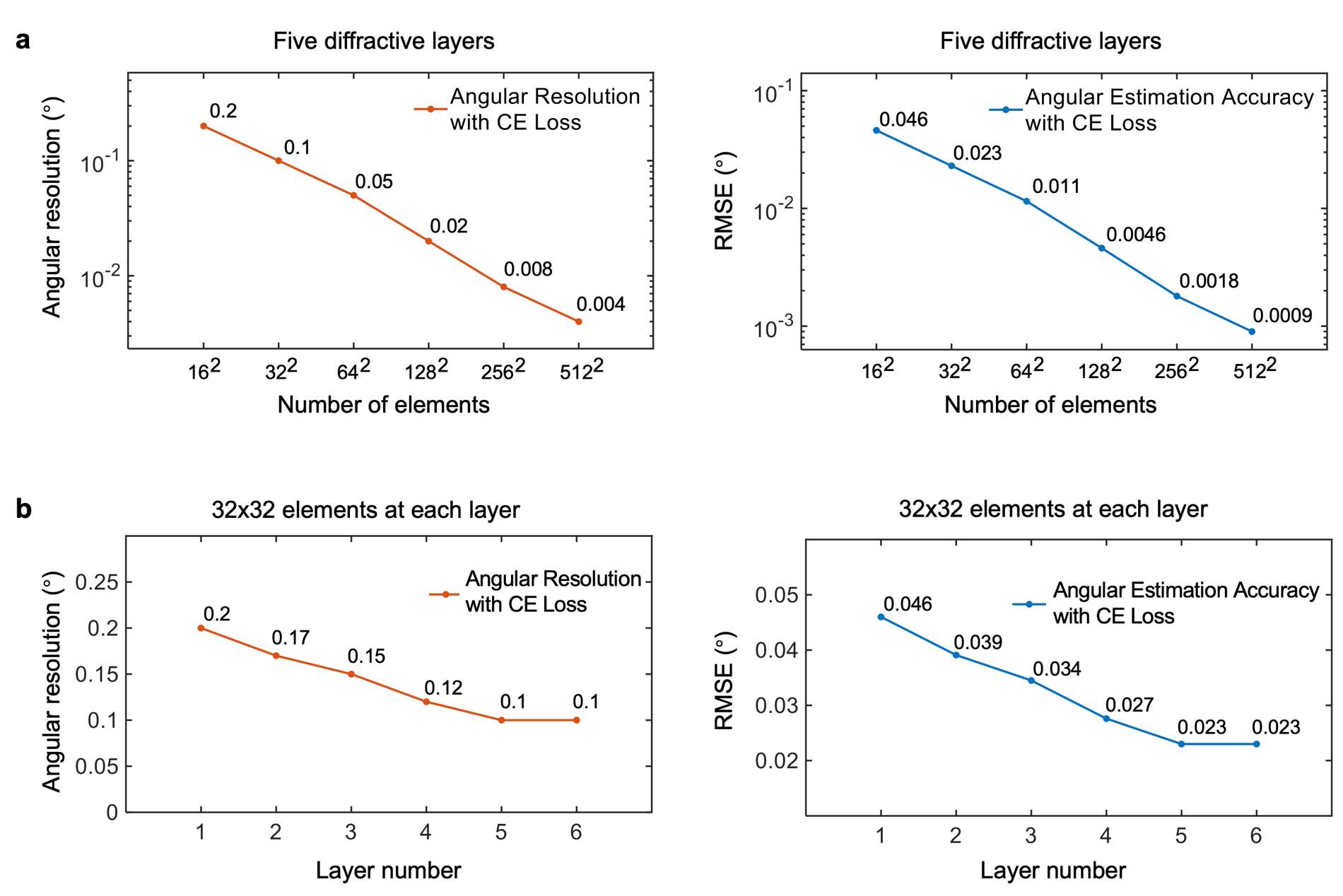


**Figure S7. Evaluating the performance upper bound by training S-DNN with CE loss function.** The evaluations of angular resolution and estimation accuracy with respect to the element numbers at each layer (**a**) and layer number (**b**) are demonstrated.


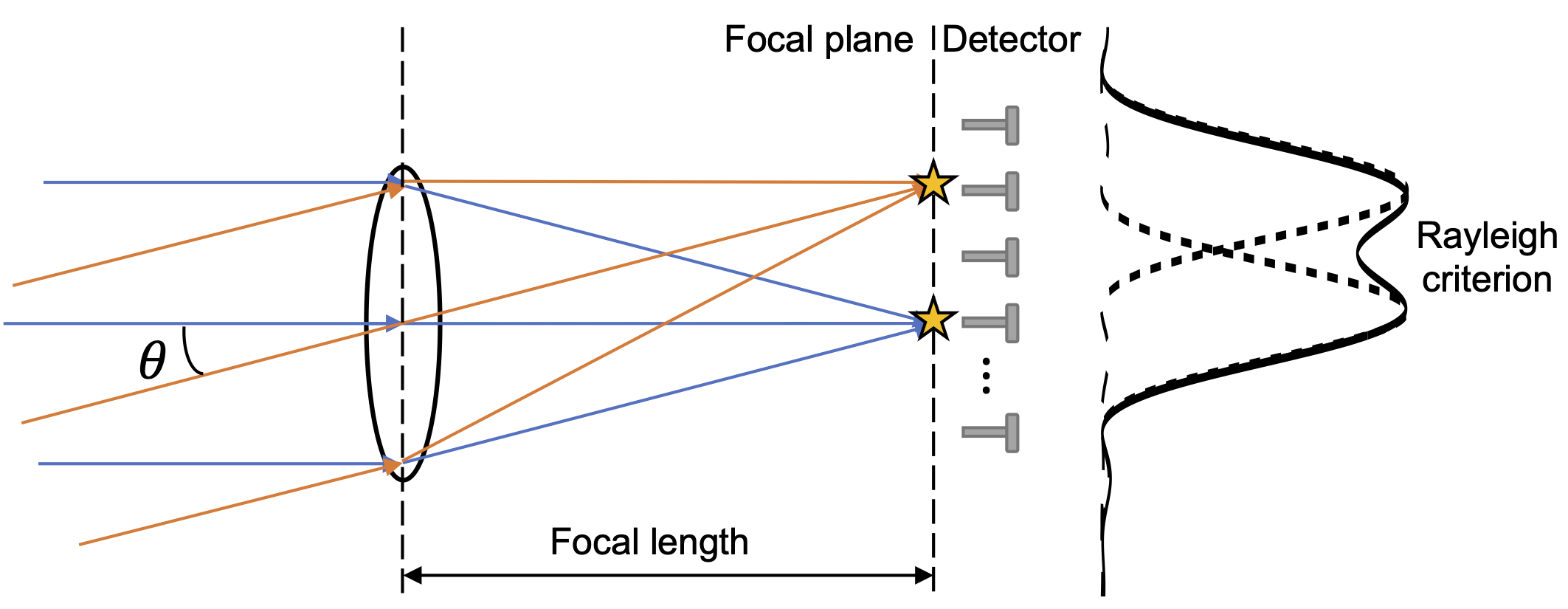


**Figure S8. Lens system for evaluating the elevation angular response.** With the same system settings with respect to the S-DNN, the elevation angular responses of the S-DNN and lens systems are evaluated within the angular range of $\left[ -5^{\circ},5^{\circ} \right]$.


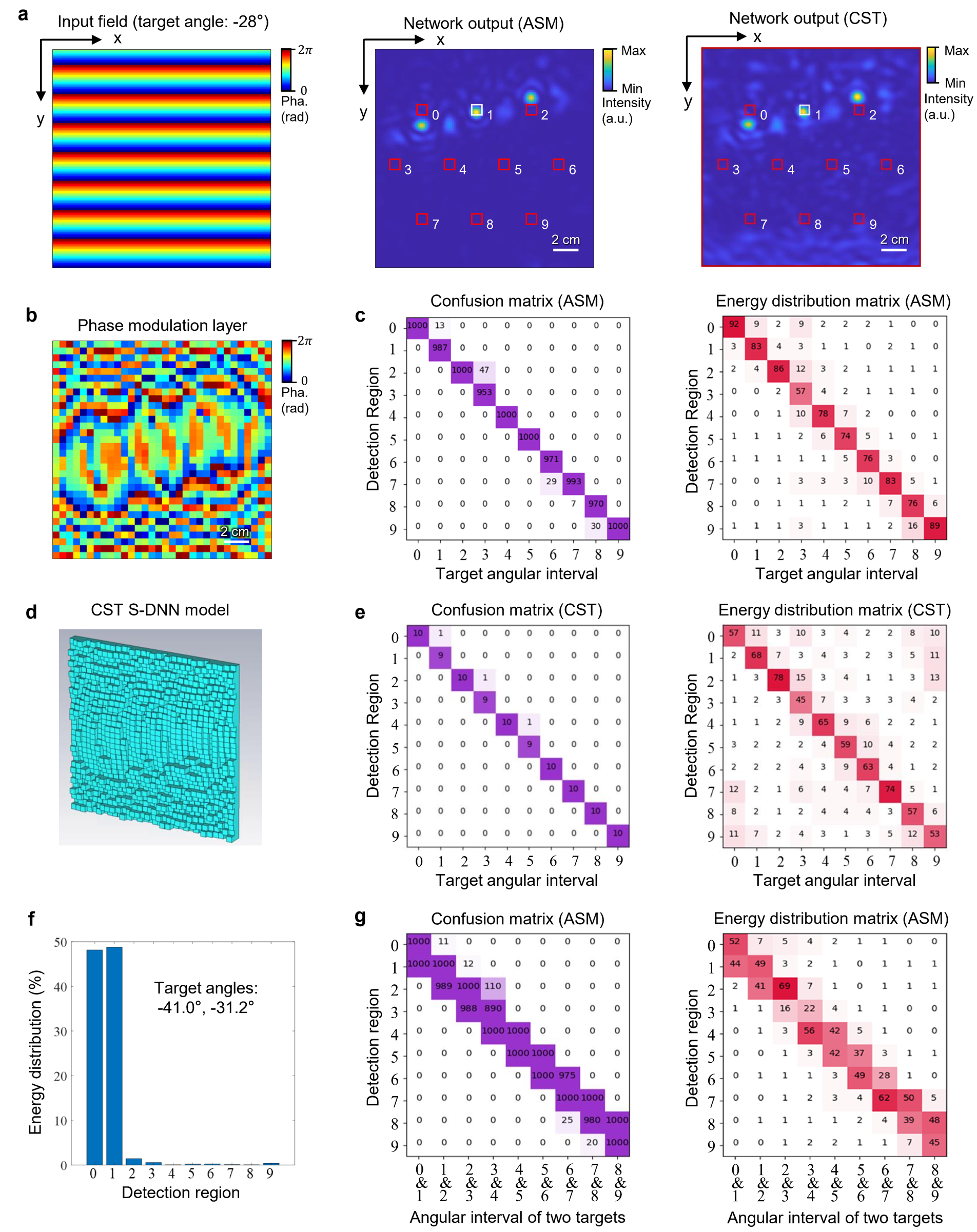


**Figure S9. Numerical results of a single-layer S-DNN for DOA estimation with** $\boldsymbol{10^{\circ}}$ **angular resolution. a**, The exemplar inference result verified with the ASM and CST numerical model of a single input target with an elevation angle of $-28.0^{\circ}$. **b**, The trained phase modulation layer of a single-layer S-DNN for the DOA estimation with $10^{\circ}$ angular resolution. **c**, The confusion and energy distribution matrices of the model evaluated on the testing dataset of a single input target with 10,000 testing samples. **d**, The CST model of single-layer S-DNN with passive layer. **e**, The confusion matrix and energy distribution matrix of the four-layer S-DNN model, evaluated by using CST simulation. **f**, The output energy distribution of a two-target testing sample with the elevation angle of $-41.0^{\circ}$ and $-31.2^{\circ}$. **g**, The confusion and energy distribution matrices of the model evaluated on the two-target testing dataset with 9,000 testing samples.

**
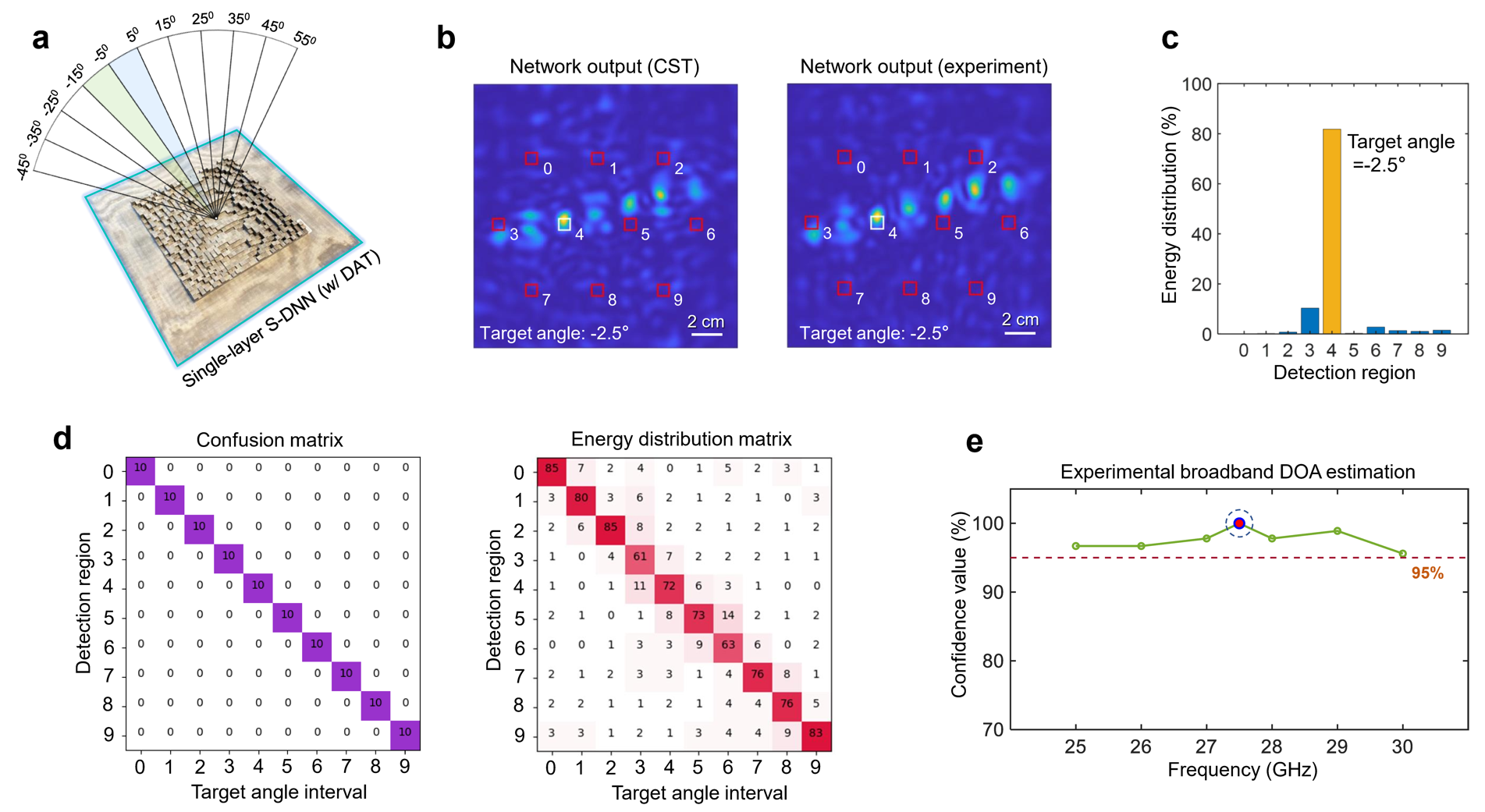
**

**Figure S10. Additional experimental results of a single-layer S-DNN for DOA estimation with** $\boldsymbol{10^{\circ}}$ **angular resolution. a**, The fabricated passive S-DNN with $10^{\circ}$ angular resolution over the angular range of $[-45^{\circ},55^{\circ}]$. **b**, The CST evaluated and experimental network outputs of a single input target with an elevation angle of $-2.5^{\circ}$. **c**, The experimental energy distribution histogram of detection regions. **d**, Experimental confusion and energy distribution matrices of S-DNN with a single input target summarized over 100 testing samples at the frequency of 27.5 GHz. **e**, Experimental DOA estimation over the broadband frequency range between 25 GHz and 30 GHz. The confidence value of each frequency point is evaluated with 100 testing samples.


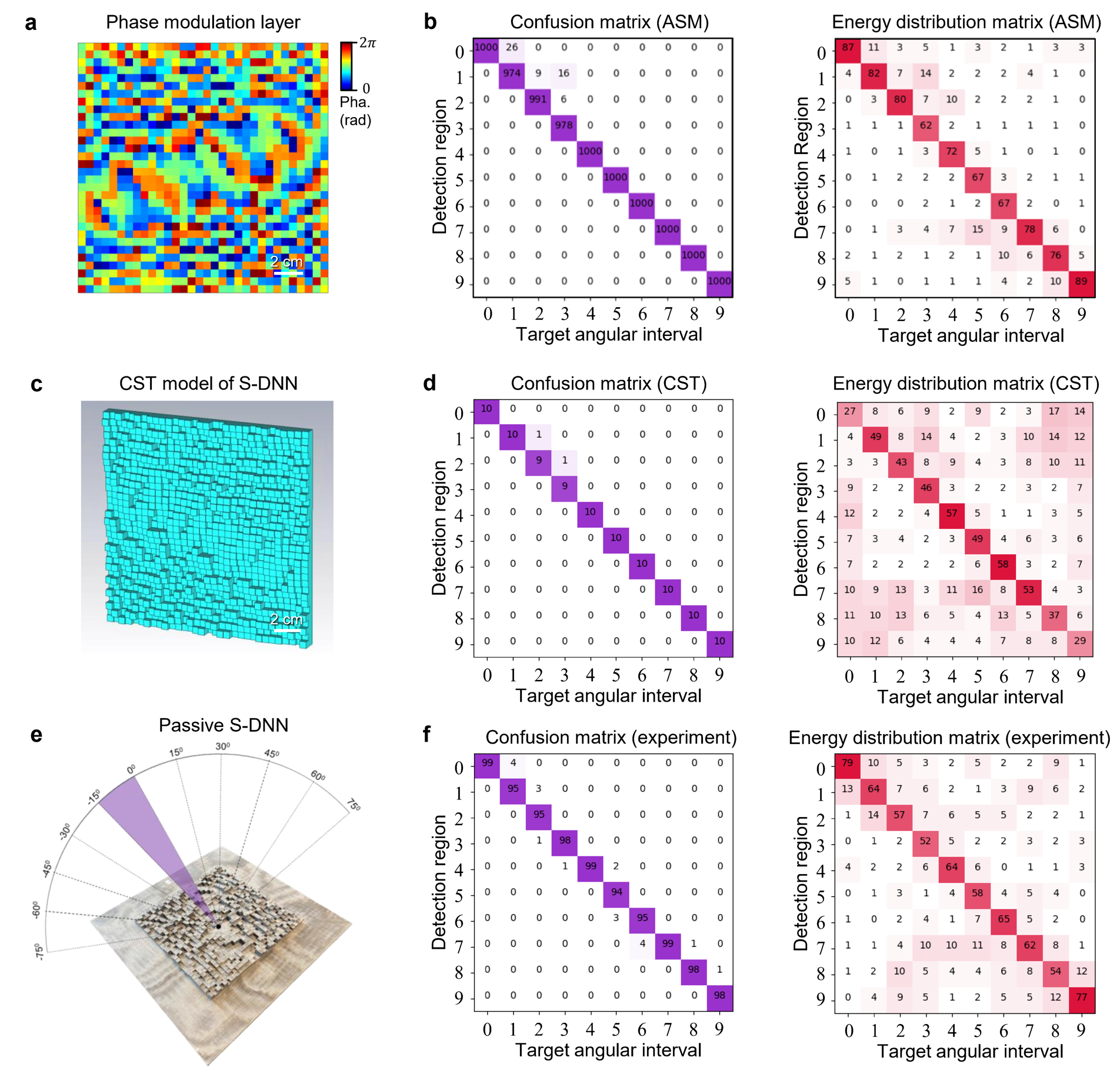


**Figure S11. Experimental and numerical results of single-layer S-DNN for single target DOA estimation with a field-of-view of** $\boldsymbol{150^{\circ}}$**.** **a**, The phase pattern of single-layer S-DNN with a field-of-view of $150^{\circ}$ and $15^{\circ}$ angular interval. **b**, The confusion and energy distribution matrices of the S-DNN evaluated on 10,000 testing samples with different angles. **c**, CST model of S-DNN. **d**, The confusion and energy distribution matrices with CST simulations evaluated on 100 testing samples. **e**, The fabricated passive S-DNN. **f**, The experimental confusion and energy distribution matrices measured on 990 testing samples.


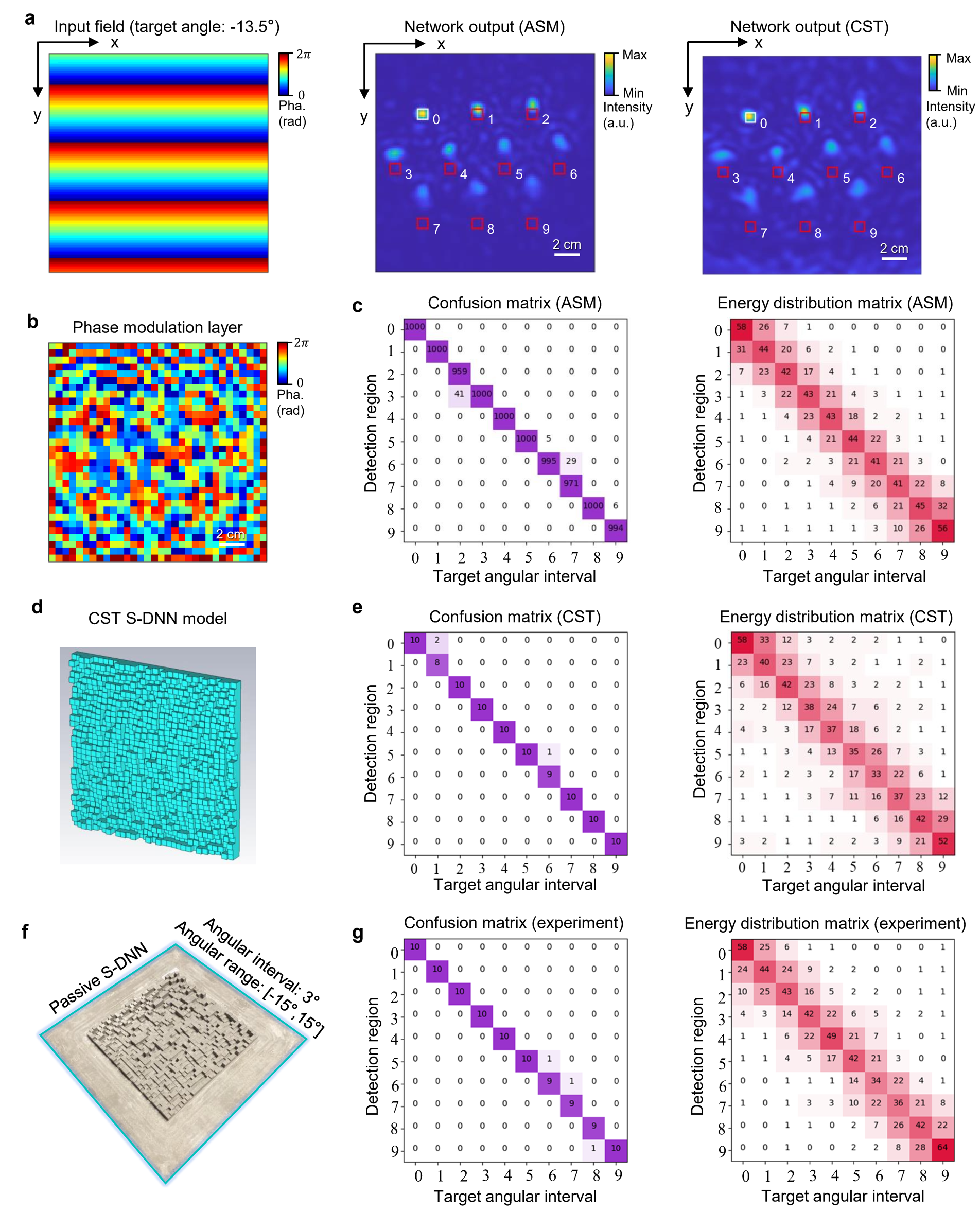


**Figure S12. Experimental and numerical results of single-layer S-DNN for single target DOA estimation with a field-of-view of** $\boldsymbol{30^{\circ}}$**. a**, The exemplar inference result verified with the ASM and CST numerical model of a single input target with an elevation angle of $-13.5^{\circ}$. **b**, The trained phase modulation layer of a single-layer S-DNN for the DOA estimation of a single target with $3^{\circ}$ angular interval. **c**, The confusion and energy distribution matrices of the model with ASM evaluations on the testing dataset of a single input target with 10,000 testing samples. **d**, CST model of S-DNN. **e**, The confusion and energy distribution matrices of the model with CST evaluations. **f**, The fabricated passive S-DNN. **g**, The experimental confusion and energy distribution matrices.


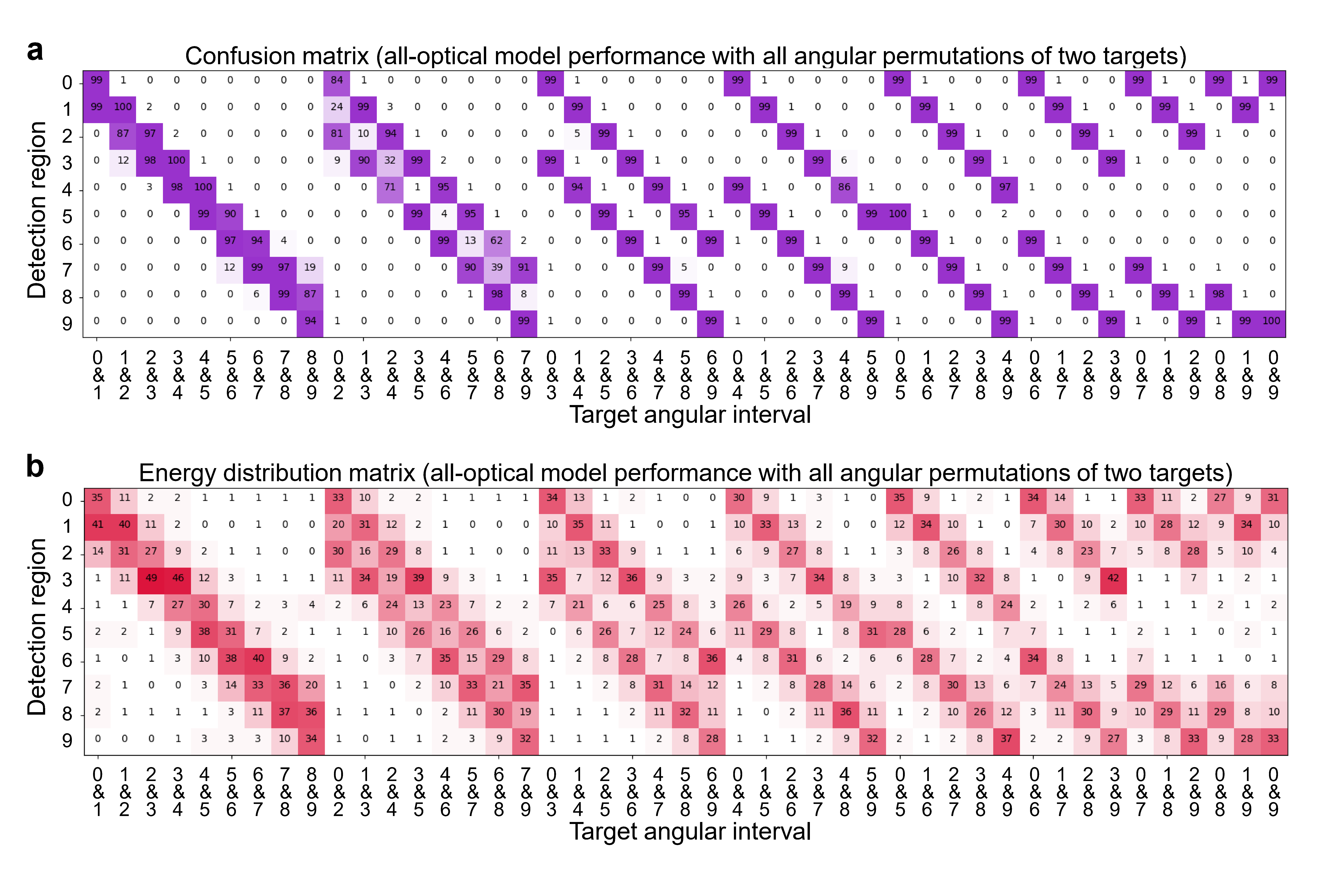


**Figure S13. Numerical results of single-layer S-DNN for the DOA estimation of two targets at arbitrary angular intervals with** $\boldsymbol{4^{\circ}}$ **angular resolution in the angular range of** $\left[ \boldsymbol{-20^{\circ},20^{\circ}} \right]$**.** The confusion matrix (**a**) and energy distribution matrix (**b**) of the model is evaluated on the two-target testing dataset that comprises 4500 testing samples. Each testing sample includes two targets distributed at arbitrary angular intervals within the angle range of $\left[ -20^{\circ},20^{\circ} \right]$. There are 45 types of angular interval permutations, each containing 100 samples with random angle combinations of two targets.


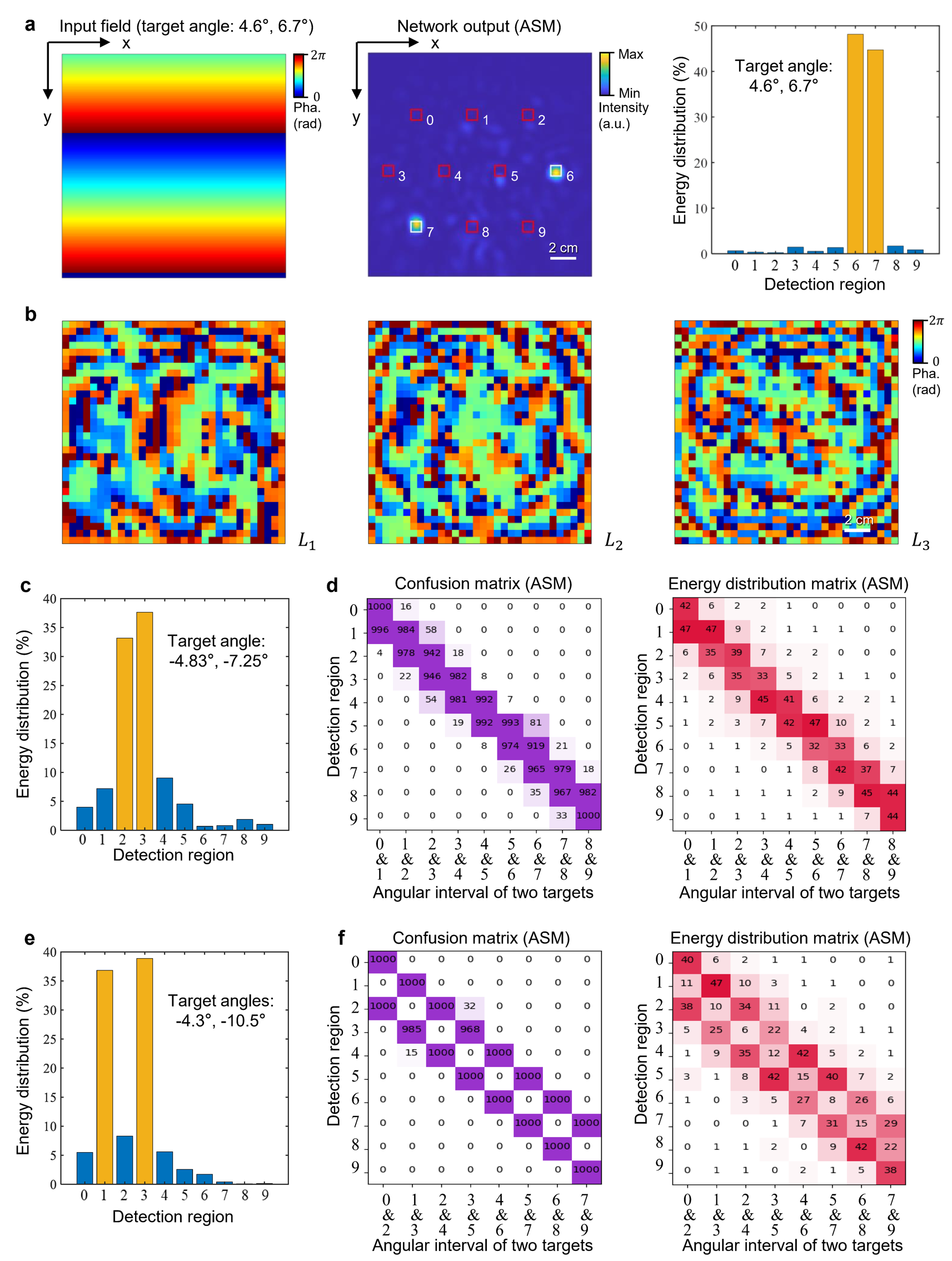


**Figure S14. Numerical results of three-layer S-DNN for the DOA estimation of two targets with** $\boldsymbol{3^{\circ}}$ **angular resolution. a**, The exemplar inference result of a two-target testing sample with the elevation angles of $4.6^{\circ}$ and $6.7^{\circ}$. **b**, The trained phase modulation layer of the broadband S-DNN for the multi-target DOA estimation with $3^{\circ}$ angular resolution. **c**, The output energy distribution of a two-target testing sample with the elevation angles of $-4.83^{\circ}$ and $-7.25^{\circ}$ at the adjacent angular intervals. **d**, The confusion and energy distribution matrices of the model evaluated with ASM numerical simulation on 9,000 testing samples. Each sample includes two targets distributed at the adjacent angular interval. **e**, The output energy distribution of a two-target testing sample with the elevation angles of $-4.3^{\circ}$ and $-10.5^{\circ}$ at two angular intervals separated by one angular interval. **f**, The confusion and energy distribution matrices of the model evaluated with ASM numerical simulation on 8,000 testing samples. Each sample includes two targets at two angular intervals separated by one angular interval.


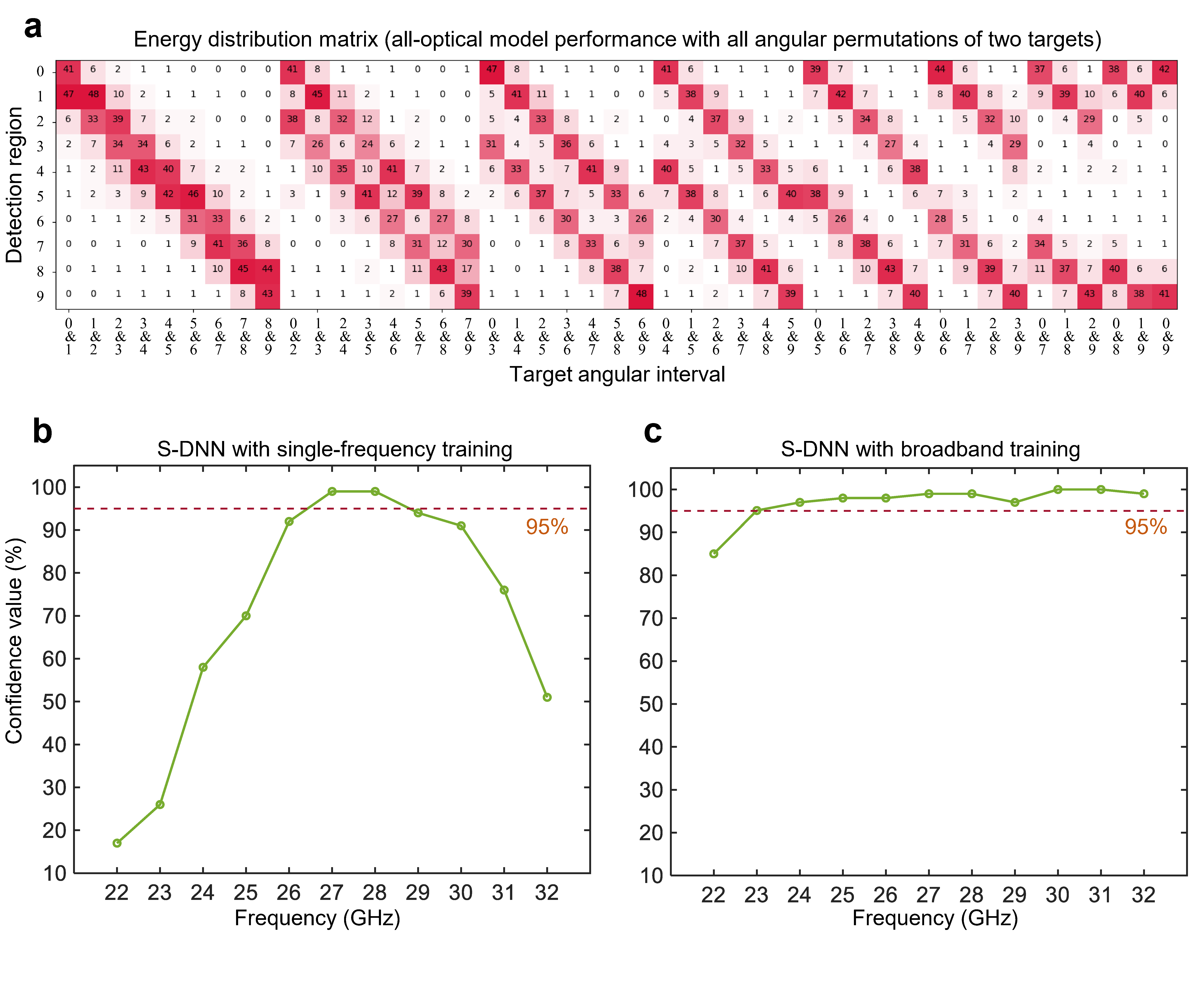


**Figure S15. Numerical results of three-layer S-DNN for the DOA estimation of two targets at arbitrary angular intervals with** $\boldsymbol{3^{\circ}}$ **angular resolution. a**, The energy distribution matrix of the model is evaluated on the two-target testing dataset that comprises 4500 testing samples. Each testing sample includes two targets distributed at arbitrary angular intervals within the angle range of $\left[ -15^{\circ},15^{\circ} \right]$. There are 45 types of angular interval permutations, each containing 100 samples with random angle combinations of two targets. **b**, The bandwidth of three-layer S-DNN with 3° angular resolution under the frequency range of 22 GHz ~32 GHz using a single-frequency training at 27.5 GHz and a broadband training (**c**), respectively.





**Figure S16. Flexible decision boundary on testing datasets with complete angular sampling for four-layer all-optical S-DNN.** Both numerical and experimental testing results with the fixed decision boundary have large numbers of misclassification angles around the angular interval boundaries (**a** and **b**), which can be effectively addressed with the flexible decision boundary to improve the model’s confidence value (**c** and **d**). The power ratio is calculated by sequentially comparing the top-two detected power values of the adjacent angular intervals.


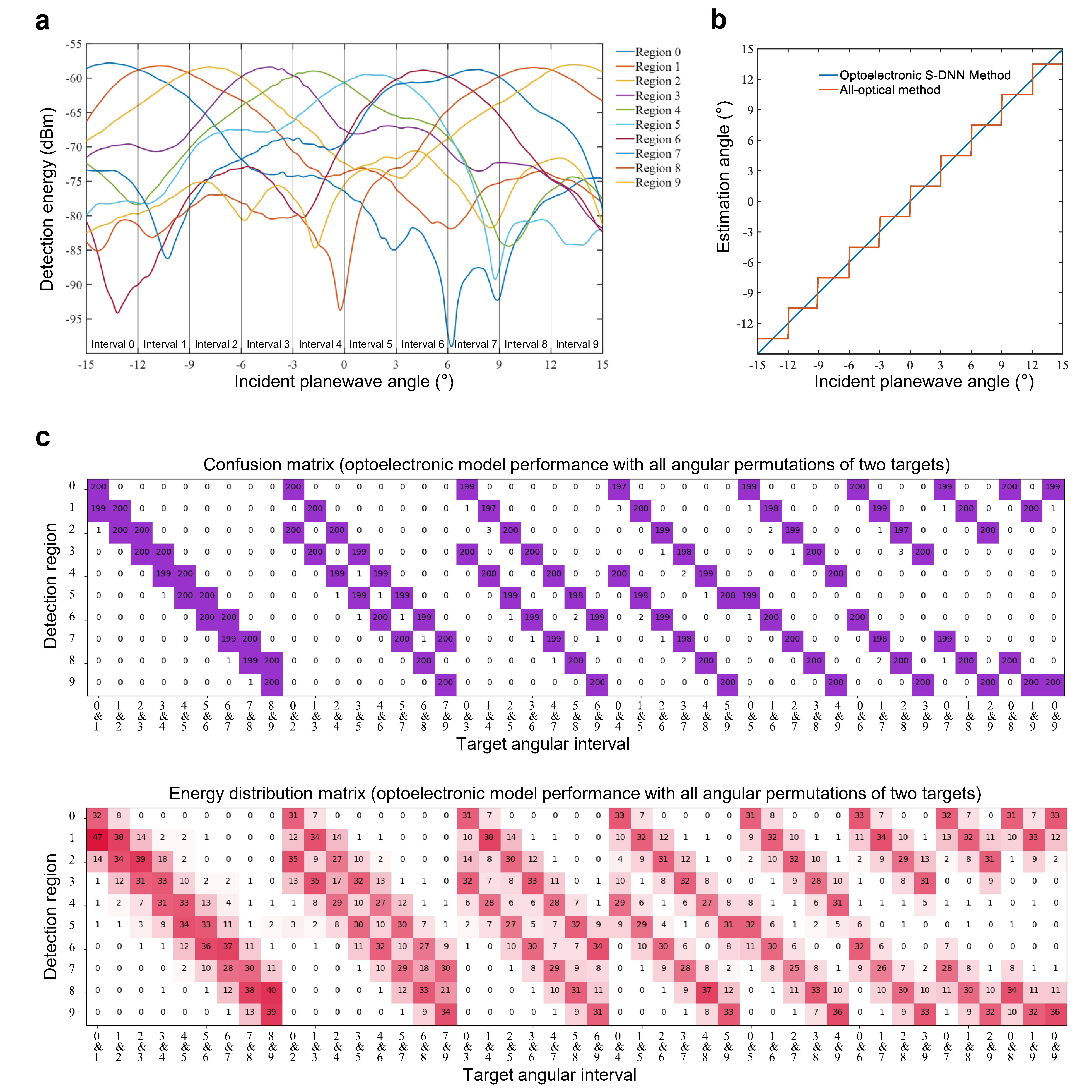


**Figure S17. High-performance DOA estimation using optoelectronic architecture for three-layer S-DNN**. **a**, The energy-angle characteristic plots of S-DNN depict the energy response of different detection regions with respect to incident plane waves at different angles. Each detection region corresponds to an angle interval of incident plane waves. **b**, The experimental comparison of the angle estimation results of optoelectronic S-DNN and all-optical S-DNN. **c**, The confusion and energy distribution matrices of the model evaluated on the two-target testing dataset based on the optoelectronic S-DNN architecture. The test sample includes two targets distributed at the arbitrary angular intervals within the angle range of $\left[ -15^{\circ},15^{\circ} \right]$, and there are a total of 45 permutations of different angular intervals, each containing 200 random angles.


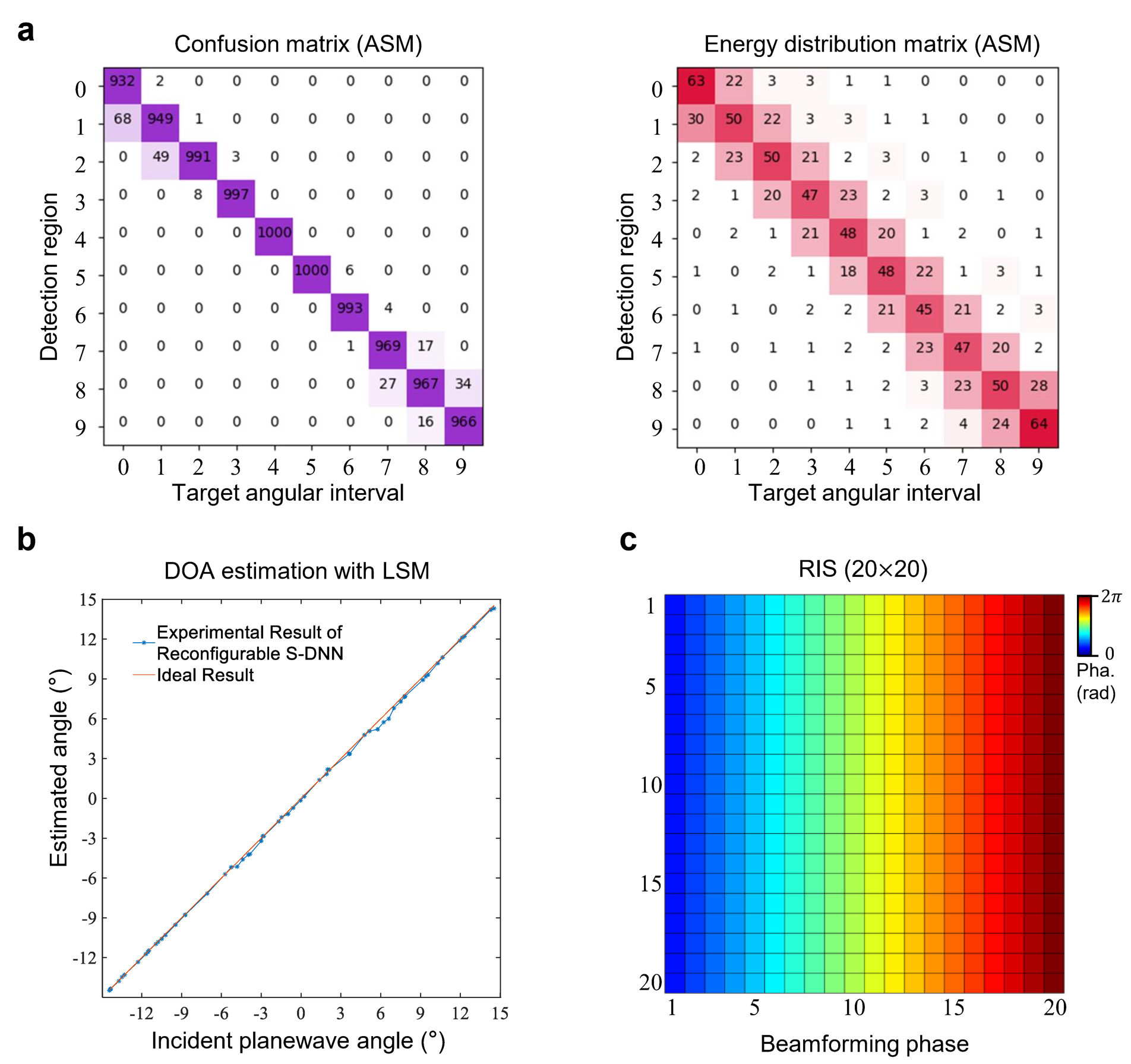


**Figure S18. Experimental results of DOA estimation and beamforming using reconfigurable S-DNNs**. **a**, The confusion and energy distribution matrices of the reconfigurable S-DNN evaluated on the testing dataset of a single input target with 10,000 testing samples. **b,** Experimental DOA estimation results of reconfigurable S-DNN based on the optoelectronic method within the angle range of $\left[ -15^{\circ},15^{\circ} \right]$. **c**, The optimized beamforming phase of the reconfigurable SDNN enables the reflection of the transmitted wave from the base station with an input angle of $-7^{\circ}$ to the user with an output angle of $12^{\circ}$.

**
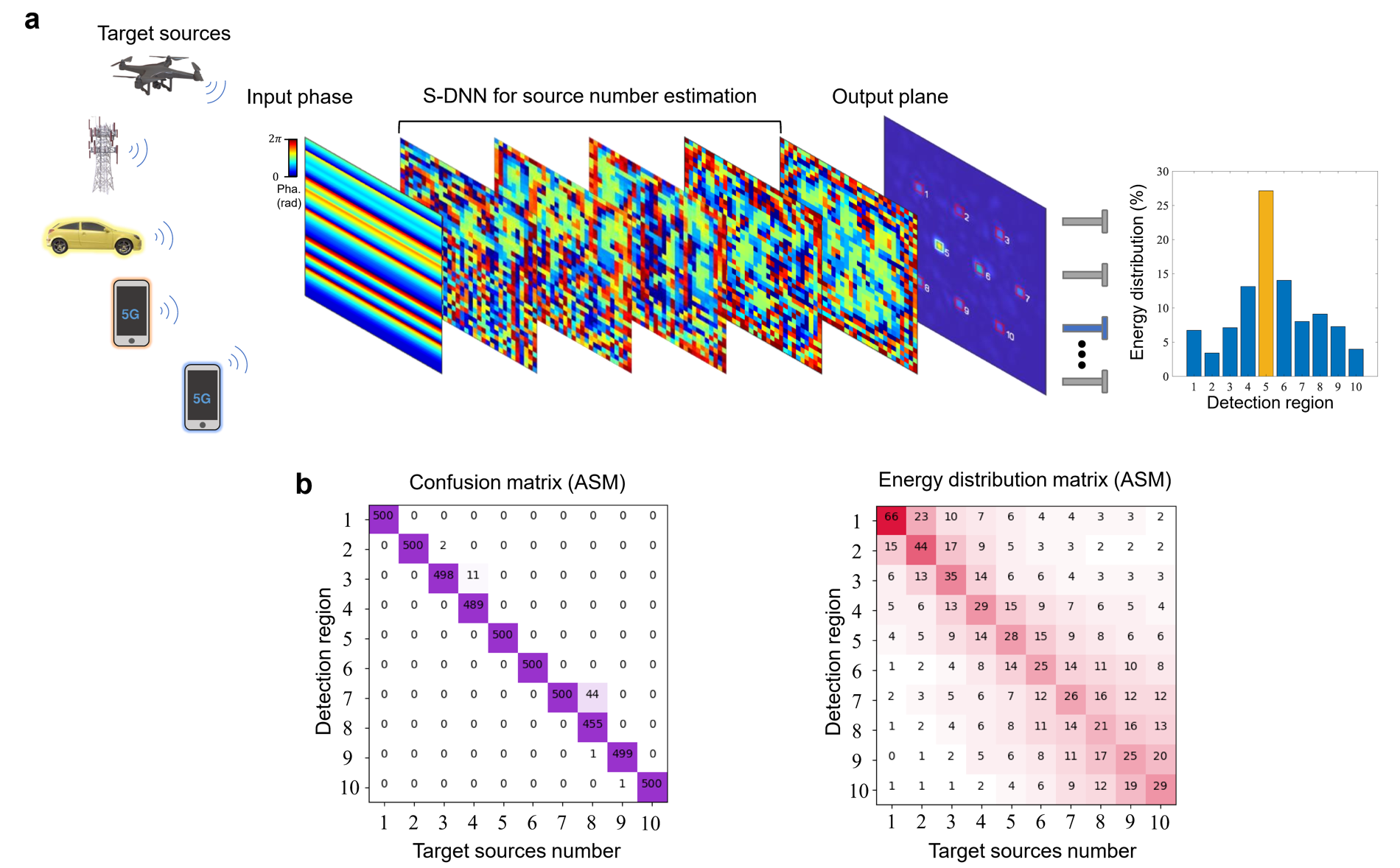
**

**Figure S19. All-optical source number estimation based on the diffractive neural networks**. **a**, The principle of S-DNN for source number estimation, where an exemplar inference results of five target sources with different angular intervals show the No. 5 detection region has the highest energy value, i.e., the number of target sources is five. The input EM field is superimposed by five complex-value EM fields from each target. **b**, The confusion and energy distribution matrices of the S-DNN evaluated on the testing dataset include 5,000 multi-target samples, and the number of targets ranges from 1 to 10.


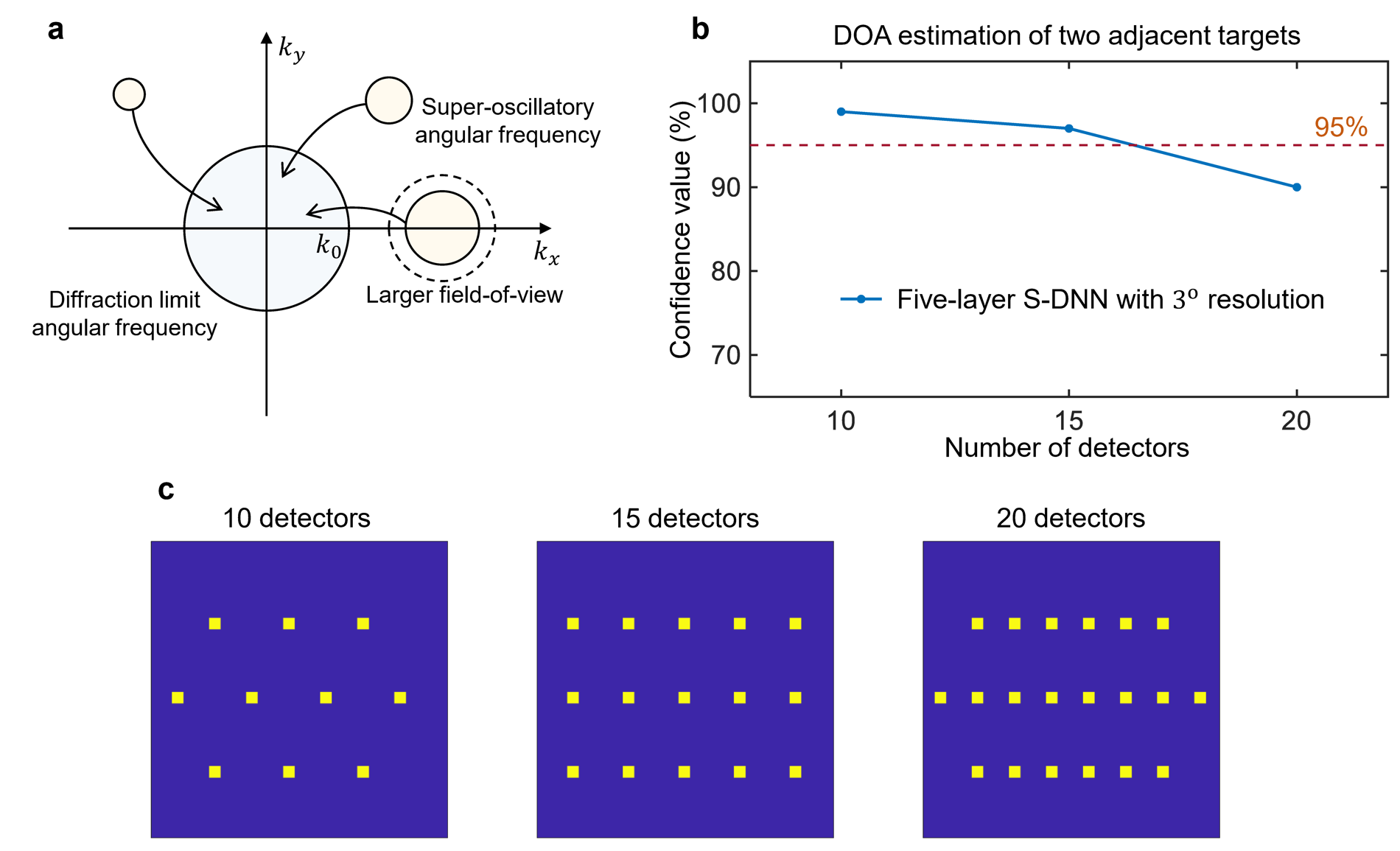


**Figure S20. The upper bound field-of-view size of S-DNN. a,** Explaining the super-oscillatory angular response of S-DNN based on angular frequency movement. **b**, The DOA estimation performance of five-layer S-DNN with 3° resolution for two sources with respect to the number of detection regions. **c**, Arrangement of different numbers of detection regions on the output plane.


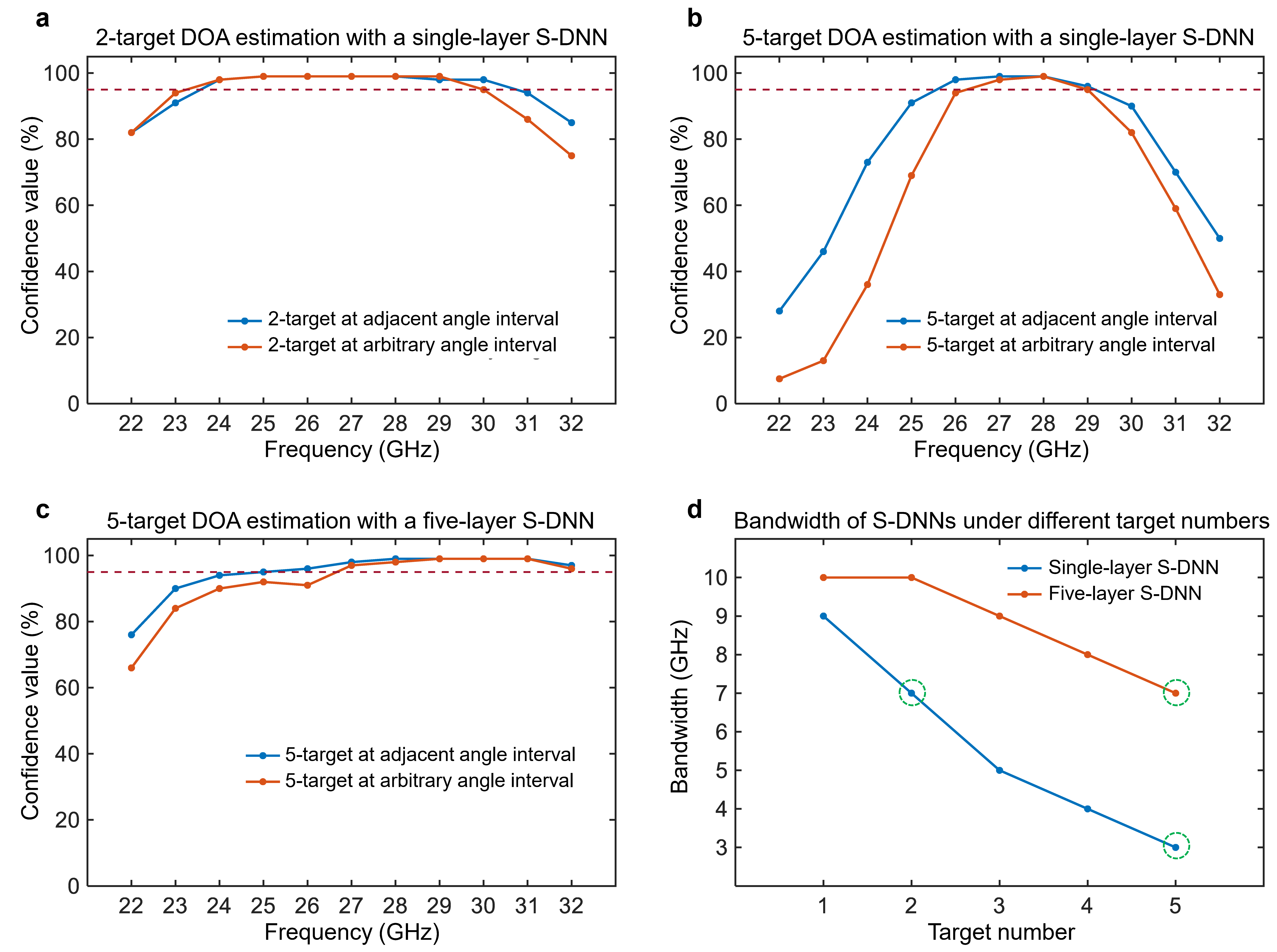


**Figure S21. Bandwidth of single-layer and multi-layer S-DNNs under different target numbers.** The broadband DOA estimation performance of a single-layer S-DNN for two target sources (**a**) and five target sources (**b**) in an angular range of [-45°, 55°]. **c**, The broadband DOA estimation performance of a five-layer S-DNN for five target sources. **d**, The bandwidth of a single-layer S-DNN and a five-layer S-DNN under different target numbers.

**References**

[s1] Wood, R. W. Physical Optics. (New York: The MacMillan Company, 1919).

[s2] Du, Y. et al. Electrically tunable liquid crystal terahertz phase shifter driven by transparent polymer electrodes. *Journal of Materials Chemistry C*. **4**, 4138–4142 (2016).

[s3] Mazlout, S. et al. Comparative study of two array configurations for 2D-DOA estimation in LS-MIMO systems. In *Proceedings of the 2017 Sixth International Conference on Communications and Networking (IEEE,* 2017), pp. 1–6.

[s4] Wax, M. et al. Detection of the number of coherent signals by the MDL principle. *IEEE Transactions on Acoustics, Speech, and Signal Processing.* **37**, 1190–1196 (1989).

[s5] Schmidt, R. Multiple emitter location and signal parameter estimation. *IEEE Transactions on Antennas and Propagation* **34**, 276-280 (1986).

[s6] Zhou, T. K. et al. Large-scale neuromorphic optoelectronic computing with a reconfigurable diffractive processing unit. *Nature Photonics* **15**, 367-373 (2021).
